# Transcriptomic signatures of severe acute mountain sickness during rapid ascent to 4,300 m

**DOI:** 10.3389/fphys.2024.1477070

**Published:** 2025-01-29

**Authors:** Ruoting Yang, Aarti Gautam, Rasha Hammamieh, Robert C. Roach, Beth A. Beidleman

**Affiliations:** ^1^ Medical Readiness Systems Biology, Walter Reed Army Institute of Research, Silver Spring, MD, United States; ^2^ Altitude Research Center, University of Colorado Anschutz Medical Center, Aurora, CO, United States; ^3^ Military Performance Division, United States Army Research Institute of Environmental Medicine, Natick, MA, United States

**Keywords:** NGS - next generation sequencing, acute mountain sickness, biomarker, machine learning, high altitude

## Abstract

**Introduction:**

Acute mountain sickness (AMS) is a common altitude illness that occurs when individuals rapidly ascend to altitudes ≥2,500 m without proper acclimatization. Genetic and genomic factors can contribute to the development of AMS or predispose individuals to susceptibility. This study aimed to investigate differential gene regulation and biological pathways to diagnose AMS from high-altitude (HA; 4,300 m) blood samples and predict AMS-susceptible (AMS+) and AMS-resistant (AMS─) individuals from sea-level (SL; 50 m) blood samples.

**Methods:**

Two independent cohorts were used to ensure the robustness of the findings. Blood samples were collected from participants at SL and HA. RNA sequencing was employed to profile gene expression. Differential expression analysis and pathway enrichment were performed to uncover transcriptomic signatures associated with AMS. Biomarker panels were developed for diagnostic and predictive purposes.

**Results:**

At HA, hemoglobin-related genes (HBA1, HBA2, and HBB) and phosphodiesterase 5A (PDE5A) emerged as key differentiators between AMS+ and AMS− individuals. The cAMP response element-binding protein (CREB) pathway exhibited contrasting regulatory patterns at SL and HA, reflecting potential adaptation mechanisms to hypoxic conditions. Diagnostic and predictive biomarker panels were proposed based on the identified transcriptomic signatures, demonstrating strong potential for distinguishing AMS+ from AMS− individuals.

**Discussion:**

The findings highlight the importance of hemoglobin-related genes and the CREB pathway in AMS susceptibility and adaptation to hypoxia. The differential regulation of these pathways provides novel insights into the biological mechanisms underlying AMS. The proposed biomarker panels offer promising avenues for the early diagnosis and prediction of AMS risk, which could enhance preventive and therapeutic strategies.

## Introduction

Acute mountain sickness (AMS) is a common altitude illness associated with rapid ascent to altitudes ≥2,500 m in unacclimatized lowlanders (Roach et al., 2002). Headache is the cardinal symptom of AMS and may be accompanied by malaise, poor appetite or nausea, dizziness, and, in some individuals, sleep disturbances (Richalet et al., 2021). AMS usually appears in the first 6–12 h of altitude exposure, reaches maximum intensity after 18–24 h of exposure, and resolves over the next few days if no further ascent occurs (Beidleman et al., 2013). The incidence of AMS increases with higher elevations and faster ascent rates (Meier et al., 2017). Approximately 10%–25% of people suffer from AMS around 2,500 m, but symptoms are usually mild and unlikely to affect daily activities (Honigman et al., 1993). However, AMS can occur in 50%–90% of individuals between 3,500 m and 5,500 m and symptoms can be debilitating such that individuals cannot perform the easiest of tasks (Beidleman et al., 2013; Hackett et al., 1976; Zafren et al., 2017). Severe AMS can also progress to life-threatening high-altitude pulmonary edema (HAPE) and high-altitude cerebral edema (HACE), both of which are life-threatening if left untreated (Honig et al., 1992).

Under identical ascent conditions to the same elevation, some individuals remain well while others develop AMS. Known risk factors that contribute to the development of AMS include rapid ascent, younger age, lack of acclimatization, and prior history of AMS and/or migraine (Beidleman et al., 2019; Luks et al., 2017; Richalet et al., 2012; Roach et al., 2000; Schneider et al., 2002). Genetic factors may also contribute to the development of AMS or play a predisposing role in an individual’s susceptibility to AMS (MacInnis and Koehle, 2016). Recent studies have identified single nucleotide polymorphisms (SNPs) that may be adaptive to high-altitude environments. Specific polymorphisms of hypoxia-inducible factor-2 alpha (HIF2α), and Egl nine homolog 1 (ENGL1) genes, which are involved in the hypoxia inducible factor-1alpha (HIF-1α) oxygen sensing pathways, have demonstrated either a higher or lower frequency in those that succumb to AMS (Buroker et al., 2012; Yasukochi et al., 2018; Yu et al., 2016; Zhang et al., 2014).

Studies have shown that changes in gene expression can be predictive of AMS. For example, exposure to high altitude has been linked to increased expression of *HMGB1, TRL4*, and *LY96* (genes associated with inflammation) and *ANGPTL4*(which inhibits *VEGF* expression) (Pham et al., 2022; Goodin et al., 2013). Conversely, *IL10* (an anti-inflammatory cytokine) was downregulated in individuals susceptible to AMS (Liu et al., 2017).

This study explores the differences in gene expression between individuals susceptible to acute mountain sickness (AMS) and those resistant. The study aims to identify genes that are differentially regulated at sea level and high altitude in those with AMS compared to those without, and to assess if these genes could predict AMS susceptibility before ascent or diagnose it at high altitude. Lastly, the study investigates the molecular pathways involved in AMS susceptibility. To achieve the study’s goal, peripheral blood mononuclear cells (PBMCs) were collected at sea level and high altitude from two independent research studies. This approach aimed to reduce the risk of spurious findings and enhance the validity of the study. The data from one study (Pikes Peak cohort) served as a discovery set, while data from the second study (Chamber cohort) was used as a validation set. The researchers hypothesized that ascent to high altitude would increase AMS symptomatology and alter transcriptomic profiles, especially the inflammation and immune system related pathways, and that a subset of differentially expressed genes could predict and diagnose AMS.

## Materials and methods

### Ethical approval

The study was approved by the Institutional Review Boards at the US Army Research Institute of Environmental Medicine (USARIEM) and the Human Research Protection Office, US Army Medical Research and Materiel Command, Ft. Detrick, MD. All volunteers provided written and verbal acknowledgement of their informed consent and were made aware of their right to withdraw without prejudice at any time. Investigators adhered to the policies for protection of human subjects as prescribed in Department of Defense Instruction 3,216.02 and the research was conducted in adherence with 32 Code of Federal Regulations Part 219 on the use of volunteers in research.

### Study volunteers

As well as 10 healthy volunteers (n = 2 women, n = 8 men) exposed for 24-h to 4,300 m in the USARIEM hypobaric chamber (Pb = 460 *mmHg*) (Figueiredo et al., 2022).

### Study design

#### Pikes Peak study

The study was part of a larger randomized, single-blind study conducted over a 30-day period (Beidleman et al., 2017). After 4 days of baseline testing at SL (50 m, 756 Torr), volunteers were flown from USARIEM to Colorado Springs, Colorado (2,000 m), where they spent the remainder of the day and night breathing supplemental oxygen (O_2_). The peripheral oxygen saturations (SpO_2_) were continuously monitored, and the mean values were maintained at 97 ± 2% from their time of arrival at 2,000 m until their time of departure for the summit the next morning. The next morning, volunteers were removed from supplemental O_2_ at 0600, driven to the summit of Pikes Peak (4,300 m, 460 Torr) and arrived around 0800. Fasting resting venous blood samples were drawn at the same time of day (0700) at SL and after 24-h exposure to 4,300 m. This cohort of volunteers was utilized as the training data set.

The study included 18 healthy volunteers (n = 5 women, n = 13 men) exposed for 24-h to 4,300 m on the summit of Pikes Peak (Pb = 460 *mmHg*) (Beidleman et al., 2017). All volunteers were healthy, well nourished, and physically active, with hematologic and ferritin values in the normal range, in age range from 18 to 39 years. None had been diagnosed with a sleep disorder, heart, or pulmonary condition. All were born at altitudes <1,500 m and none had lived at altitudes >1,200 m in the previous 3 months prior to the start of the study.

#### Chamber study

The study was part of a larger randomized, single-blind study conducted over a 14-day period (Figueiredo et al., 2022). After 4 days of baseline testing at SL (50 m, 756 mmHg), volunteers ascended over a 15-min period to 4,300 m (460 Torr) in a hypobaric chamber. Following the initial 15-minute ascent and a rest period, all volunteers underwent 3 h of walking exercise (40 min on and 20 min off) on a treadmill conducted at ∼40% of SL peak oxygen uptake (V̇O_2peak_) to simulate light exercise on/off under controlled environmental (20°C ± 2°C; 40% ± 5%) conditions. The fasting, resting blood samples were obtained at the same time of day (0700) at SL and after 24-h exposure to 4,300 m. This cohort of volunteers was used as the test data set to validate results from the training data set. One volunteer was dropped from the Chamber study due to an unrelated medical problem after ∼6 h of exposure and prior to AMS symptoms, and two individuals dropped due to severe AMS after ∼12 h of exposure. Thus, no HA blood sample was collected on these individuals. One volunteer completed the Chamber study, but the SL blood sample did not pass quality control, so only their AMS score and HA blood sample were used in the analyses.

Physical and demographic characteristics were determined at SL and are presented in Table 1.

**TABLE 1:** Physical characteristics of volunteers at sea level (SL) and physiologic characteristics at high altitude (HA).

Study	Sex (M/W)	Age (Years)	BMI (kg/m^2^)	Height (cm)	Weight (kg)	V̇O_2peak_ (ml⋅kg^−1^⋅min^−1^)	SpO_2_ (%)	HR (bpm)	AMS-C
Pikes Peak 4,300 m (*N* = 18)	13/5	23 ± 6	23.2 ± 2.8	179 ± 10	76.9 ± 13.3	45.9 ± 5.4	84.7 ± 5.4	90.8 ± 11.8	1.3 ± 1.1
Chamber 4,300 m (*N* = 10)	8/2	24 ± 6	24.0 ± 2.7	173 ± 12	72.6 ± 12.3	45.5 ± 4.6	84.8 ± 4.7	93.4 ± 5.5	1.8 ± 1.1

Means ± Standard Deviation (SD); M/W, men and women; BMI, body mass index; V̇O_2peak_, peak oxygen uptake, SpO_2_, pulse arterial oxygen saturation, HR, heart rate; AMS-C, Acute mountain sickness cerebral factor score.

### Research procedures

#### Body weight (BW) and height

The BW while wearing long pants and t-shirts without shoes was measured every morning of the study (Seca 9070, Seca, Inc. Chino, CA). Body height was measured once at SL using a stadiometer (Seca 217, Seca, Inc., Chino, CA).

#### Peak oxygen uptake ( $V^{˙}$ O_2peak_)

An incremental, progressive exercise bout to volitional exhaustion on a treadmill (Model: TMX425C, Trackmaster, Newton, KS) was used to assess peak oxygen uptake (V̇O_2peak_) during the baseline SL phase of the study. Measurements of V̇E, V̇O_2_, and V̇CO_2_ were obtained using a metabolic cart (True Max 2400; Parvo Medics, Sandy, UT) calibrated with certified gases and volume standard. Volunteers walked for a total of 10 min (5 stages) starting at 3 mph/0% grade and ending at 4 mph/7% grade. The treadmill speed and grade were then changed to 6 mph and 0%, respectively for 2 min. Then, every 2-min stage thereafter, the speed and/or grade was increased slightly until O_2_ uptake failed to increase or the volunteer could no longer continue despite encouragement (11).

#### Acute mountain sickness (AMS)

The incidence and severity of AMS was determined from the shortened version of the Environmental Symptoms Questionnaire (ESQ) (Beidleman et al., 2007; Sampson et al., 1983) at least 1 hour after any exercise bout. AMS questionnaires were administered at SL and after 24-h exposure to 4,300 m in both studies. The cerebral factor score (AMS-C) was calculated from the ESQ and if AMS-C was ≥1.4 (Sampson et al., 1983), the individual was considered to have severe AMS. Immediately after completion of the questionnaire, resting heart rate (HR) and pulse arterial oxygen saturation (SpO_2_) were assessed with a finger pulse oximeter (Model 8600; Nonin Medical Inc. Minneapolis, MN) for 2 min and the mean for each was calculated.

### Gene expression

The detailed methodology for PBMC separation and RNA sequencing is provided in Supplementary File S1. The raw count of RNA seq data was profiled using CLC Genomics Workbench (Qiagen, Germantown, MD). The samples with more than 20% low read counts (raw count <10) were filtered. In the remaining samples, the low read counts were considered as missing values, which were imputed by K-Nearest Neighbor method; however, the imputed data constitutes only a small fraction of the overall dataset. Trimmed mean of M values (TMM) normalization was used to normalize the RNA seq count using edgeR R package (Robinson et al., 2010). The principal component analysis showed no large batch effect between time points and no outliers were identified.

### Statistical analyses

Statistical analyses were performed using R version 3.6.0. Descriptive statistics were calculated for demographic characteristics and clinical AMS assessments. Biological biomarkers and continuous variables were summarized with means ± standard deviations (SD) and independent t-tests. Categorical variables were summarized with frequencies and χ^2^ tests. To evaluate group differences between phenotypes and time points, a multiple linear regression model was used, adjusting for sex as a covariate. This analysis was conducted using the edgeR R package. Given that BMI is correlated with sex and age is unrelated to AMS in this data, only sex was included as a covariate in the linear model. The small sample size of the dataset potentially leads to underestimate the dispersion, thus higher prior.df was used.

#### Classification

The Gini score was employed to rank genes based on their importance in distinguishing between AMS+ and AMS- individuals. The Gini score measures the impurity reduction achieved by splitting the samples based on gene expression values. For classification, a linear Support Vector Machine (SVM) algorithm was trained using various combinations of top-ranked genes. The performance of the SVM models was evaluated using the Area Under the Receiver Operating Characteristic (ROC) curve (AUC). AUC provides a measure of the classifier’s ability to discriminate between positive and negative instances. Additionally, the Youden index was used to determine the optimal threshold for classification, balancing sensitivity and specificity. The Youden index helps identify the threshold that maximizes the true positive rate while minimizing the false positive rate.

#### Pathway analysis

All the data analysis was conducted under R version 3.6.0. Ingenuity Pathway Analysis (IPA) (v 01-08, Qiagen, Redwood City, www.ingenuity.com) was used to determine functional pathway enrichment analysis. The statistical significance was defined by p-value <0.01. Pathways of cancers and other unrelated diseases were removed from the database.

## Results

### Blood samples

**FIGURE 1:**
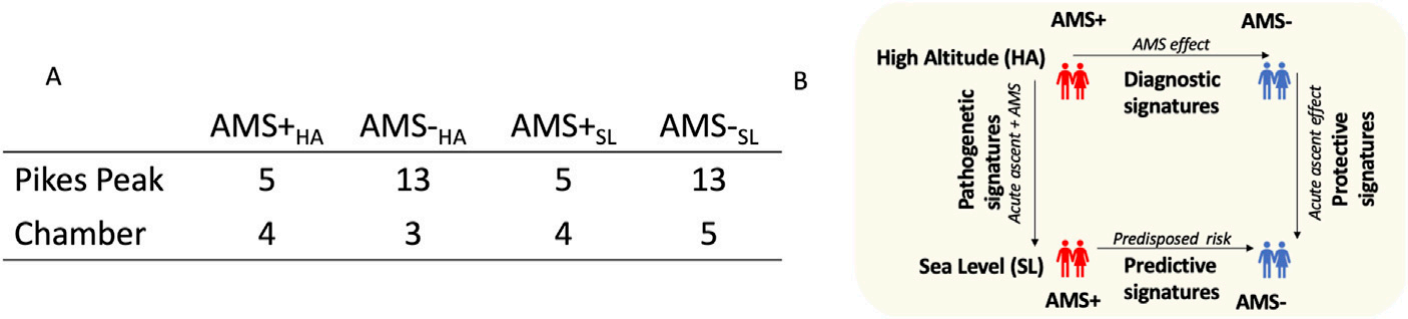
**(A)** The individuals in this study consisted of two groups; those susceptible to acute mountain sickness (AMS+) and those resistant to AMS (AMS-) with samples collected both at sea level (SL) and high altitude (HA) such that there were four groups of blood samples (AMS + _HA,_ AMS─_HA,_ AMS + _SL,_ and AMS─_SL_). **(B)** Differential genomic analyses were performed between these four groups of samples to identify biomarkers for various purposes; diagnosis, prediction, pathogenesis, and protection from AMS.

The individuals in this study consisted of two cohorts; those susceptible to acute mountain sickness (AMS+) and those resistant to AMS (AMS─) with samples collected both at sea level (SL) and high altitude (HA) in each cohort such that there were four groups of blood samples. The number of blood samples available for analysis is listed in Figure 1A.

### AMS measures

In the Pikes Peak study, 5 of 18 individuals (∼28%) were diagnosed with severe AMS following ascent to 4,300 m. In the Chamber study, 5 out of 10 individuals (∼50%) were diagnosed with severe AMS. The baseline characteristics at SL for age, height, weight, BMI, V̇O_2peak_ and percentage of women in the Pikes Peak and Chamber cohorts did not differ between cohorts (Table 1). In addition, the SpO_2_, HR, and AMS-C score measured after 24 h at HA did not differ between cohorts (Table 1).

### Differentially expressed genes (DEGs)

As illustrated in Figure 1B, our primary focus was on the identification of diagnostic and predictive biomarker panels, fine-tuned to discern between AMS+ and AMS─ individuals. First, we identified a panel of diagnostic DEGs that differed between AMS + vs. AMS─ individuals following ascent to HA (AMS + _HA_ vs. AMS─_HA_) in the same direction in both cohorts. Second, we identified a panel of predictive DEGs that differed between AMS + vs. AMS─ individuals prior to ascent to HA (AMS + _SL_ vs. AMS─_SL_) in the same direction in both cohorts. Third, we identified a panel of pathogenic DEGs in AMS + individuals going from SL to HA (AMS + _SL_ vs. AMS + _HA_) in both cohorts. Fourth, we identified a panel of protective DEGs in AMS─ individuals going from SL to HA (AMS─_SL_ vs. AMS─_HA_) in both cohorts. Last, we determined whether a subset of these DEGs trained on the Pikes Peak cohort could be used to diagnose AMS from HA samples and predict AMS from SL samples in the Chamber cohort.

#### Diagnostic biomarkers (AMS + _HA_ vs. AMS─_HA_)

**FIGURE 2:**
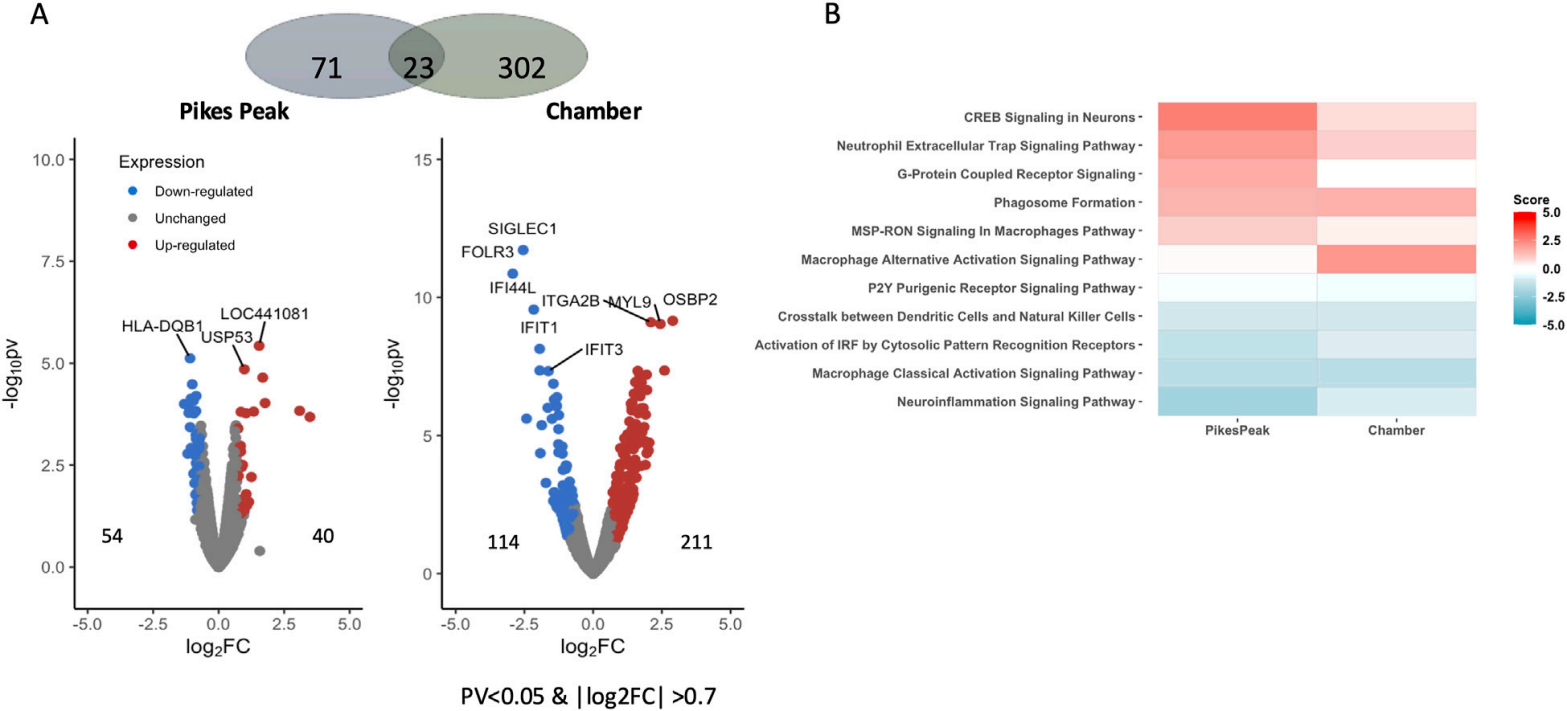
**(A)** Volcano plot shows the differentially expressed genes (DEGs) (pv < 0.05 and |Log2 Fold change| > 0.7) between AMS+_HA_ and AMS─_HA_ in the Pikes Peak and Chamber cohorts, with the x-axis representing the log fold change and the y-axis representing the −log10 p-value. The number of up- and downregulated DEGs is indicated on the side of the graph. **(B)** The heatmap shows the pathways that are commonly enriched (FDR < 0.05) and have a pathway activity score z that is not equal to zero. The red/blue color stands for the up-/downregulated pathways, respectively.

Figure 2A illustrates DEGs at HA in the two cohorts (Pikes Peak and Chamber). The Pikes Peak cohort (5 AMS+/13 AMS─) displayed 40 upregulated and 54 downregulated genes (p < 0.05, |Log2Fold change| > 0.7), while the Chamber cohort (4 AMS+/3 AMS─) displayed 211 upregulated and 114 downregulated genes using the same criteria. After false-discovery rate correction, there were 2 and 96 Benjamini–Hochberg FDR-corrected DEGs remaining for the Pikes Peak and Chamber cohorts, respectively.

Table 2 and Supplementary Figure S1 lists 11 overlapped DEGs (using the p < 0.05, |Log2Fold change| > 0.7 criteria) which exhibited the same expression profiles in both cohorts. Among the 11 common putative diagnostic biomarkers, nine genes were upregulated and nine were downregulated. Seven of these genes were reported to be associated with AMS-associated pathophysiology including hypoxic stress, inflammation, nitric oxide production and immune function (References listed in Table 2). Notably, the hemoglobin genes (*HB1* and *HB2*), which increase O_2_ affinity for Hb, were upregulated in AMS + individuals in both cohorts. Human leukocyte antitgen-DQB1 (*HLA-DQB1*), a major player in the innate immune response, was downregulated in AMS + individuals in both cohorts. The pathway enrichment analysis of the two cohorts showed a good overlap (Figure 2B; Supplementary Table S1). In detail, several DEGs in the pathway analysis were related to inflammation proteins. *NF* κ *B2* and *STAT2* were downregulated in AMS + individuals in both cohorts and statistically significant in one of the cohorts, diminishing their roles in suppressing immune responses, while *IL1R2*, a type 2 interleukin 1 (IL-1) receptor that negatively regulates IL-1 inflammation signaling, was significantly overexpressed in AMS + individuals in both cohorts (Supplementary Table S1). G-protein coupled receptor (GPCR) signaling, and a range of neuroinflammation and immune responses were identified as common signaling pathways in both cohorts. The GPCR-signaling pathway was activated, especially Phosphodiesterase 5A (*PDE5A*), which was upregulated in AMS + _HA_ in both cohorts. At the same time, the immune system signaling pathways such as macrophage classical activation and MSP RONS were deactivated in AMS + _HA_ in both cohorts.

**TABLE 2:** Eleven Common Diagnostic Acute Mountain Sickness (AMS) Differentially Expressed Genes that Occurred in the Same Direction in Both the Pikes Peak and Chamber Cohorts Following Ascent to 4,300 m High Altitude (HA) in AMS-susceptible (AMS + _HA_) and AMS-resistant (AMS─_HA_) individuals.

			Pikes Peak			Chamber		
Symbol	Description	Evidence	PV	FDR	log2FC	PV	FDR	log2FC
ISG15	ISG15 ubiquitin like modifier	Anti-Inflammation (Mirzalieva et al., 2022) ↓	1.95e-03	1	−0.851	2.38E-05	0.103	−1.260
HLA-DQB1	Major histocompatibility complex, class II, DQ beta 1	Susceptible HLA alleles (Qinggele et al., 2009)	1.08E-05	0.136	−1.092	2.11E-03	1	−0.773
LOC101927999	Putative uncharacterized protein FLJ44672		9.64E-05	1	−1.313	3.15E-03	1	−0.728
HBA2	Hemoglobin subunit alpha 2	Hypoxia (Soma, 2012) ↑	1.50E-04	1	3.091	1.53E-02	1	0.841
HBA1	Hemoglobin subunit alpha 1	Hypoxia (Soma, 2012) ↑	2.08E-04	1	3.483	2.64E-03	1	1.153
FOLR3	Folate receptor gamma	Immune (Gaur et al., 2020) HA exposure ↑	1.65E-03	1	−1.183	1.35E-11	1.69E-07	−2.923
SIGLEC1	Sialic acid binding Ig like lectin 1		4.91E-03	1	−0.959	2.08E-12	2.60E-08	−2.545
GAS6	Growth arrest specific 6	Hypoxia (Lei et al., 2022) ↓	5.28E-03	1	−0.843	1.62E-02	1	−0.861
TNNT1	Troponin T1, slow skeletal type	Hypoxia (Britto et al., 2020) ↑	2.77E-02	1	0.755	7.54E-06	9.41E-02	1.952
DRAXIN	Dorsal Inhibitory Axon Guidance Protein		3.74E-02	1	−0.818	2.05E-03	1	−1.267
PDE5A	Phosphodiesterase 5A	Nitric Oxide (Corbin et al., 2004) ↑	4.43E-02	1	0.755	1.99E-03	1	1.145

#### Predictive biomarkers (AMS + _SL_ vs. AMS─_SL_)

**FIGURE 3:**
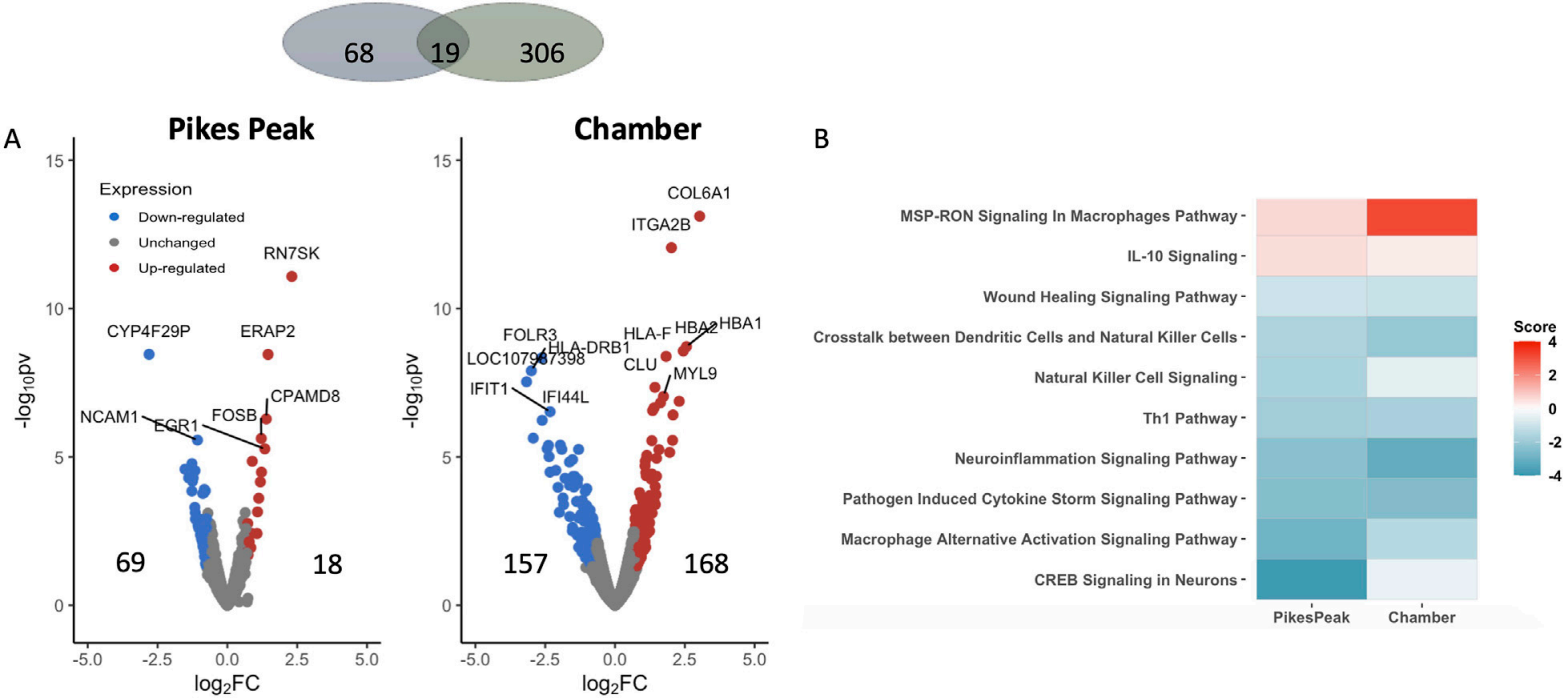
**(A)** Volcano plot shows the differentially expressed genes (DEGs) (pv < 0.05 and |Log2 Fold change | > 0.7) between AMS + _SL_ and and AMS─_SL_ in the Pikes Peak and Chamber cohorts, with the x-axis representing the log fold change and the y-axis representing the −log10 p-value. The number of up- and downregulated DEGs is indicated on the side of the graph. **(B)** The heatmap displays the pathways that are commonly enriched (FDR <0.05) and have a nonzero pathway activity. The red/blue color stands for the up-/downregulated pathways.

Figure 3A illustrates DEGs at SL in the two cohorts (Pikes Peak and Chamber). Eighteen up- and 69 downregulated genes were identified in the Pikes Peak cohort (p < 0.05, |Log2Fold change| > 0.7), while 168 up- and 157 downregulated genes were identified in the Chamber cohort. There were 14 and 60 genes that remained significant after Benjamini–Hochberg FDR correction in the Pikes Peak and Chamber cohorts, respectively.

Nineteen DEGs identified in the Pikes Peak cohort also occurred in the Chamber cohort (p < 0.05, |Log2Fold change| > 0.7) in the same direction (Table 3; Supplementary Figure S2). Among the 11 common predictive biomarkers, three genes were upregulated and eight genes were downregulated. Notably, *KIF19*, responsible for cerebrospinal fluid flow regulation in the brain. and *TNNT1*, which regulates skeletal slow-twitch muscle contraction, were upregulated in both cohorts at SL. Three DEGs exhibited the same patterns of regulation in AMS + individuals both at SL (predictive) and HA (diagnostic). For instance, *FOLR3, DRAXIN*, involved in the innate immune response and cell survival, were downregulated while *TNNT1* was upregulated in AMS + individuals in both cohorts regardless of whether the blood samples were taken at SL or HA. The significant alterations in these genes in the same direction in AMS + individuals in both cohorts indicates that they might be regarded as genetically predisposing risk factors for AMS.

**TABLE 3:** Eleven Common Predictive Acute Mountain Sickness (AMS) Differentially Expressed Genes that Occurred in the Same Direction in Both the Pikes Peak and Chamber Cohorts at sea level (SL) in AMS-susceptible (AMS + _SL_) versus AMS-resistant (AMS─_SL_) individuals.

			Pikes Peak			Chamber		
Symbol	Description	Evidence	PV	FDR	log2FC	PV	FDR	log2FC
DUSP2	Dual Specificity Phosphatase 2	Hypoxia (Hsiao et al., 2017) ↓	1.51E-05	1.89E-01	0.890	3.45E-03	1	0.760
HLA-DQA1	Major histocompatibility complex, class II, DQ alpha 1	Susceptible HLA alleles (Cao et al., 2022)	2.71E-05	3.39E-01	−1.161	5.68E-03	1	−0.957
COL5A3	Collagen Type V Alpha 3 Chain	Hypoxia (Jadaun et al., 2022) ↑	3.20E-05	4.00E-01	−1.510	1.06E-02	1	−0.921
TNNT1	Troponin T1, slow skeletal type	Hypoxia (Britto et al., 2020) ↑	3.65E-05	4.57E-01	1.176	1.07E-07	1.34E-03	2.077
KIF19	Kinesin Family Member 19		1.37E-04	1	1.128	2.20E-04	1	1.447
C1QB	Complement C1q B Chain	Immune (Schmitz et al., 2022)	6.71E-04	1	−1.158	3.76E-02	1	−0.935
		HA exposure ↑						
DDX11L2	DEAD/H-Box Helicase 11 Like 2		1.03E-03	1	−1.004	2.86E-03	1	−1.352
ZNF595	Zinc Finger Protein 595		1.25E-03	1	−0.778	8.33E-03	1	−0.988
DRAXIN	Dorsal Inhibitory Axon Guidance Protein		1.11E-02	1	−0.836	1.29E-02	1	−1.096
FOLR3	Folate Receptor Gamma	Immune (Gaur et al., 2020)	2.15E-02	1	−0.822	5.54E-09	6.94E-05	−2.635
		HA exposure ↑						
S100B	S100 Calcium Binding Protein B	HA exposure (Bjursten et al., 2010) ↑	4.05E-02	1	−0.782	1.02E-04	1	−2.041

Figure 3B and Supplementary Table S2 displays the commonly enriched pathways and associated DEGs observed in both the Pikes Peak and Chamber cohorts. Notably, immune response, neuroinflammation, wound healing, and CREB (cAMP Response Element-Binding Protein) signaling pathways were suppressed prior to ascent to HA. Additionally, seven of these pathways overlapped with the DE pathways of diagnostic biomarkers. Additionally, AMS + _SL_ individuals demonstrated an inhibition of CREB, GPCR, phagosome formation, and macrophage-alternative activation signaling in comparison to AMS─_SL_ individuals. However, upon ascent to HA, susceptible individuals exhibited a switch to activation of these four pathways. In contrast, the immune system, including neuroinflammation signaling, crosstalk between dendritic cell and natural killer cells were inhibited and macrophage pathway were activated before and after ascent and remained consistent both at SL and HA in AMS + individuals.

#### Pathogenic biomarkers (AMS + _SL_ vs. AMS + _HA_)

**FIGURE 4:**
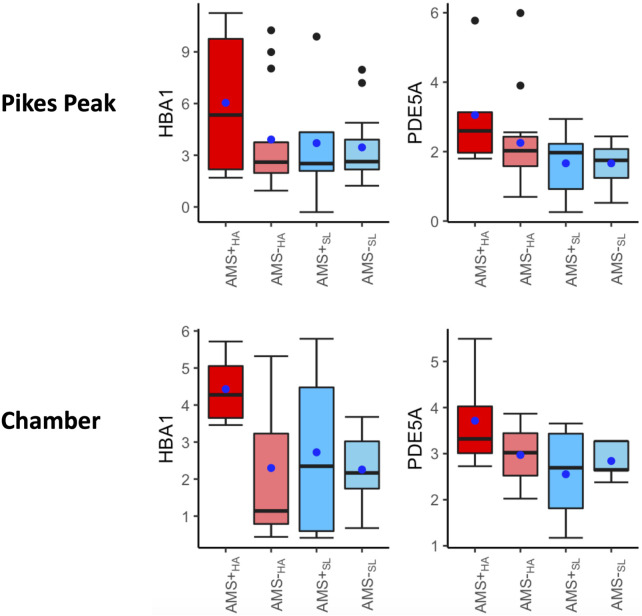
Box plots of HBA1, PDE5A, HBB in four categories: AMS + _HA_, AMS─_HA,_ AMS + _SL,_ and AMS─_SL_. The *t*-test p-values between any two categories are listed on the top of the bars, with three significance levels. Three biomarkers were significantly increased in AMS individuals in response to altitude changes, compared to the non-AMS individuals. The blue dot indicates the mean and middle bar is the median.

Supplementary Table S3 contains the DE signaling pathways and associated DEGs in AMS + individuals ascending from SL to HA. These pathogenic markers measure both the acute effect of ascent to HA and the AMS effect. Forty-nine common DEGs (32 upregulated and 21 downregulated in both cohorts) and seven common DE pathways were identified in both cohorts for AMS + individuals (Supplementary Table S3). There was a significant overlap between several DE pathways in AMS + individuals ascending from SL to HA (AMS + _SL_ vs. AMS + _HA_) and those pathways identified in diagnostic biomarkers (AMS + _HA_ vs. AMS─_HA_) (Supplementary Table S1). Specifically, CREB signaling in neurons, G-protein coupled receptor signaling and phagosome formation signaling were common in both the pathogenic and diagnostic DE pathways. Notably, two genes, HBA1, PDE5A, exhibited significant increases during acute ascent to HA in AMS + individuals (AMS + _SL_ vs. AMS + _HA_), and their expression levels were also significantly higher in AMS + individuals compared to AMS─ individuals, both at SL (AMS + _SL_ vs. AMS─_SL_) and HA (AMS + _HA_ vs. AMS─ _HA_) (Figure 4).

#### Protective markers (AMS─_SL_ vs. AMS─_HA_)

Supplementary Table S4 contains the DEGs and DE signaling pathways in AMS─ individuals ascending from SL to HA. These protective markers measure only the HA effect. Only nine common DEGs with same regulatory direction (*EGR2, HDC, HBA2, HBA1, ZNF702P, GATA2, HLA-DMB, SNORA104, CCR5AS* (6 up- and 3 downregulated) and one enriched signaling DE pathway were found in both cohorts for AMS─ individuals (Supplementary Table S4). This finding suggests that the genetic disturbance resulting from acute ascent to HA has a limited impact on AMS─ individuals but exerts a dramatic effect on AMS + individuals*.* Given that the pathways in AMS─ individuals remained relatively unchanged during ascent, it is not surprising to observe significant overlap between the majority of DE pathways in AMS + individuals ascending from SL to HA and those identified in diagnostic biomarkers (AMS + _HA_ vs. AMS─_HA_). There was a significant overlap between one DE pathway (MSP_RON Signaling in Macrophages) in AMS─ individuals going from SL to HA (AMS─_SL_ vs. AMS─_HA_) with both the diagnostic and predictive DE signaling pathways.

### Converged enriched pathways

**FIGURE 5:**
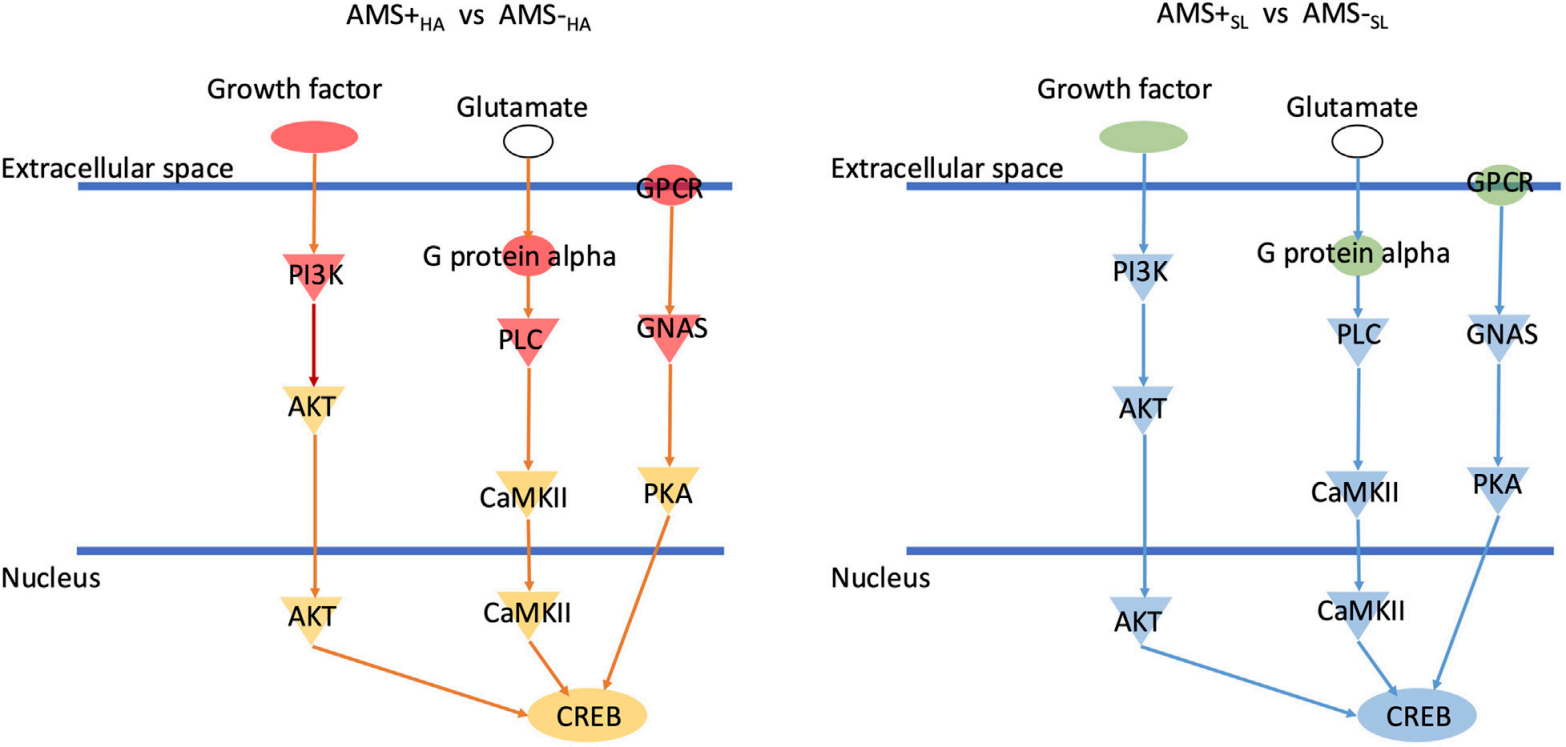
CREB signaling is downregulated in AMS + _HA_ compared to AMS- _HA_ individuals but upregulated when individuals were downregulated at sea level (AMS + _SL_ vs. AMS- _SL_). Green means downregulation and red mean upregulation. Blue network means inhibition while orange network means activation.

Combining the enriched pathways for diagnostic, predictive, and pathogenic biomarkers, only the CREB signaling pathway was significantly expressed in all categories. Interestingly, CREB signaling is upregulated in AMS + _HA_ compared to AMS─_HA_ individuals but downregulated when the same individuals were at sea level (AMS + _SL_ vs. AMS─_SL_) (Figure 5). The DEGs in the CREB pathway are focused on various receptors. Combining enriched pathways for diagnostic, predictive, and protective biomarkers, only the MSP-RON signaling pathway was significantly expressed in all categories.

### Putative biomarker panels

In this study, the Pikes Peak cohort was used as a training data set to identify biomarkers and develop machine learning algorithms capable of distinguishing AMS+ and AMS─ individuals. These algorithms were subsequently tested on the Chamber cohort test data set.

#### Putative AMS diagnostic panel

**FIGURE 6:**
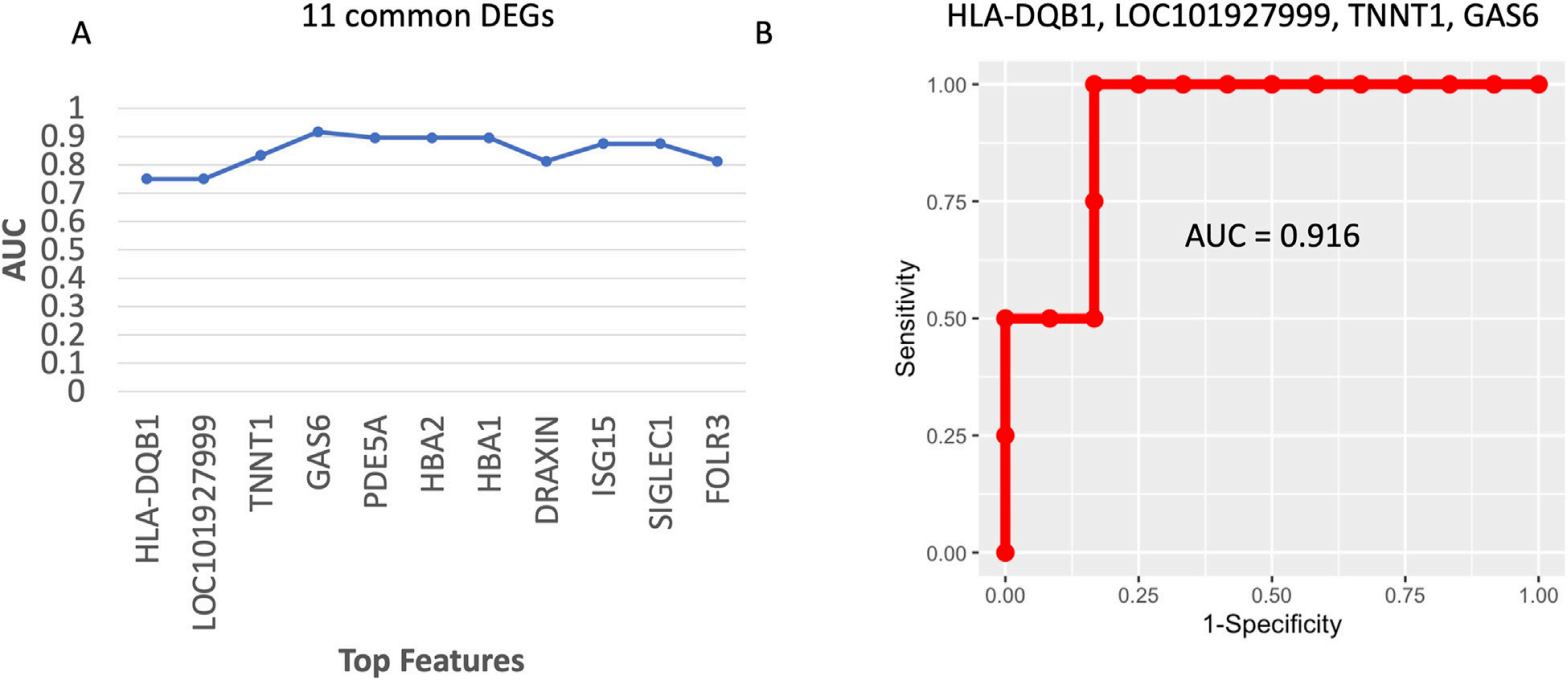
**(A)** The forward trajectory of the AUC for different gene combinations based on the 23 common DEGs in Table 2. **(B)** The ROC curve of the best 5-gene diagnostic biomarker panel.

In this study, 11 diagnostic biomarkers (Table 2) existed in common in both the Pikes Peak and Chamber cohorts. The goal was to fine tune the 11 biomarkers for future validation studies. Our model was trained using the 36 samples from the Pikes Peak cohort (18 SL samples and 18 HA samples), which consisted of 5 samples with AMS+ and 31 samples with AMS─. The Gini score, derived from a Random Forest model consisting of 10,000 trees, was used to rank the eleven genes. Subsequently, we trained linear Support Vector Machine (LSVM) classifiers on various combinations of genes, starting with the top 1 gene and progressively adding more genes (up to top N) (Figure 6A). These classifiers were then tested using 16 samples from the Chamber cohort (9 SL samples and 7 HA samples), which consisted of 4 individuals with AMS+ and 12 individuals with AMS─. Figure 6A illustrates the forward trajectory of the Area Under ROC curve (AUC) for the gene combinations. The best performance was achieved using the top 4 genes: major histocompatibility complex, class II, DQ beta 1 (*HLA-DQB1*), *LOC101927999*, growth arrest specific 6 (*GAS6*), and troponin T1, slow skeletal type (*TNNT1*), resulting in an AUC of 0.91 with sensitivity of 1.0 and specificity of 0.83 (Figure 6B). The mechanism of these genes is listed in Table 2.

#### Putative AMS predictive biomarker panel

Following the diagnostic biomarker panel approach, we initially identified 11 common DEGs between the Pikes Peak and Chamber cohorts as listed in Table 3. LSVM classifiers were trained on the Pikes Peak cohort and subsequently tested on the Chamber cohort. Among the genes ranked the highest according to the Gini score, *TNNT1* emerged as the highest-ranking gene, resulting in an AUC of 1.0, perfect sensitivity, and specificity. Notably, *TNNT1* is also in the AMS diagnostic biomarker panel.

## Discussion

This is a unique study applying next-generation sequencing to detect transcriptomic changes associated with AMS symptoms before and after rapid exposure to 4,300 m in unacclimatized lowlanders. Two independent cohorts were utilized to mitigate the risk of false results. We hypothesized that rapid ascent to 4,300 m in unacclimatized lowlanders would induce AMS in some individuals and not others and lead to differential regulation of gene expression between these two groups. In addition, we hypothesized that AMS + _HA_ individuals compared to AMS─_HA_ would have differential regulation of pathways associated with increased inflammation and depressed immune function at HA. To evaluate these hypotheses, diagnostic, predictive, pathogenic, and protective biomarkers of AMS were evaluated as well as their converged pathways.

In this study, we first identified that hemoglobin genes (*HBB, HBA1, HBA2*) were among the most significant DEGs upregulated in AMS + compared to AMS─ individuals both at SL and HA (Figure 4). These genes were also dramatically elevated from SL to HA for the AMS + individuals but not the AMS─ individuals in both the Pikes Peak and Chamber cohorts. These findings suggest that hypoxic individuals produce more hemoglobin to compensate for lower oxygen levels in the blood. This is a well-known adaptation to high altitude exposure. What is novel is that this upregulation occurs to a greater degree in AMS + individuals both at SL and HA, indicating that AMS + individuals may have a higher level of hypoxia both at SL and HA. It is known that hemoglobin is significantly elevated in highlanders (Beall, 2006) as well as chronic mountain sickness patients (Reeves and Leon-Velarde, 2004). Hemoglobin also acts as a redox regulator and as a scavenger of the gaseous mediates nitric oxide and hydrogen sulfide (H2S). The upregulation of hemoglobin often occurs in human PBMCs in critical illness (Brunyanszki et al., 2015). Furthermore, hypocapnia, a common response to high altitude, can also hinder oxygen offloading, induce vasoconstriction, potentially elevating the hemoglobin (Curley et al., 2010).

**FIGURE 7:**
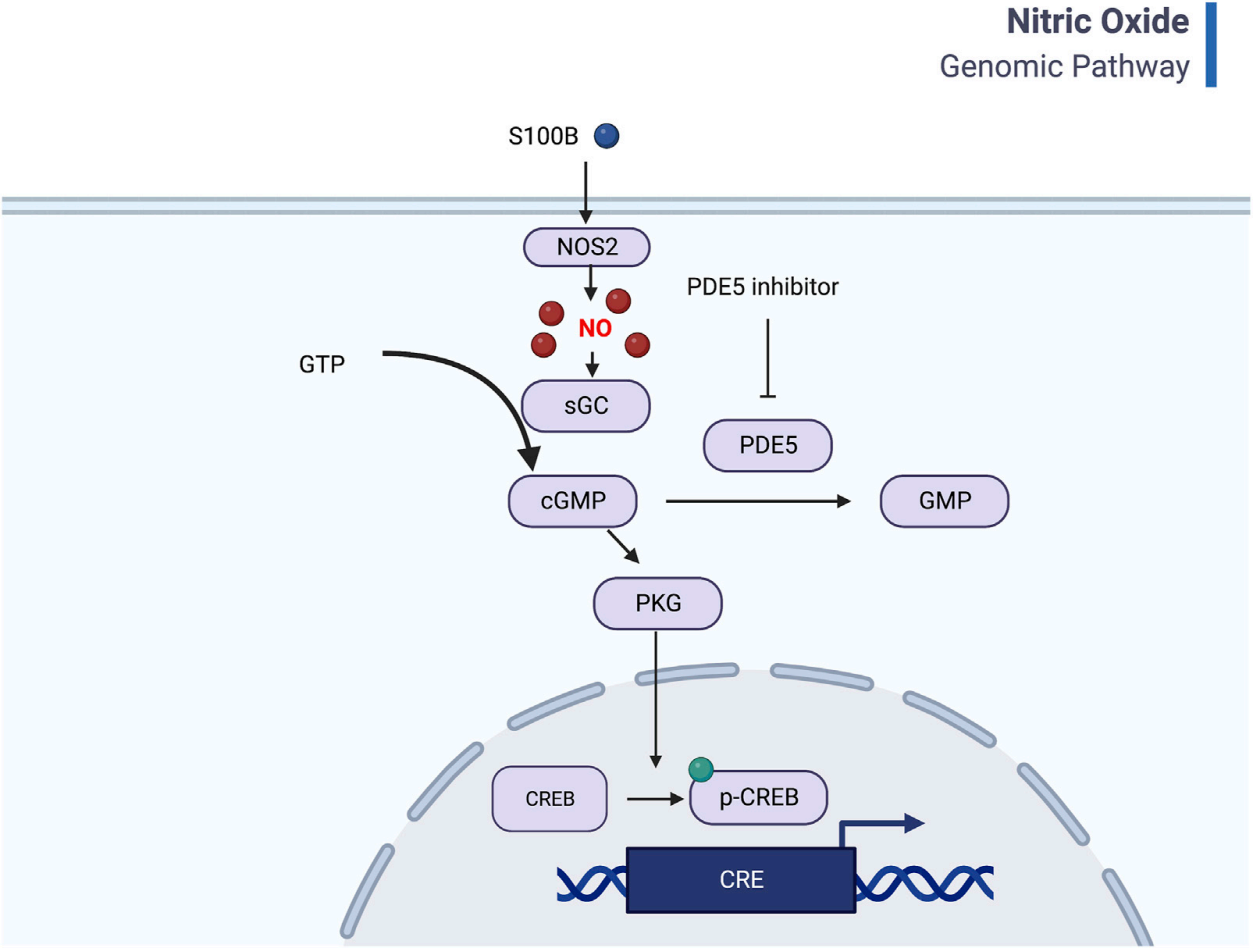
General mechanism of Nitric oxide pathway and CREB signaling. Nitric oxide binds to guanylyl cyclase and results in 3′–5′–cyclic guanosine monophosphate (cGMP) from guanosine 5′-triphosphate (GTP). Increased PDE5 will help degrade cGMP to GMP and thus lead to less cGMP-dependent protein kinase (PKG) and inhibit CREB signaling. The inflammation factors, such as S100B also regulates the NO pathway (Created with BioRender.com).

Another interesting finding was that the *PDE5A* gene was significantly upregulated between AMS+ and AMS─ in both the Pikes Peak and Chamber cohorts (Figure 4). PDE-5 is an enzyme responsible for the degradation of cyclic guanosine monophosphate (cGMP) in the lung, which ultimately reduces nitric oxide (NO) synthesis. NO is known to play a crucial role in AMS symptoms, as it triggers a cascade of events dependent on cGMP that leads to smooth muscle relaxation and a reduction in intracellular calcium. A deficiency in NO synthesis may increase the risk of AMS and high altitude pulmonary edema (HAPE) (Reeves and Leon-Velarde, 2004). Interestingly, current medications used to prevent AMS and reduce pulmonary vasopressor response, such as Sildenafil, Tdalafil, and Theophylline, are both PDE-5 inhibitors (Corbin et al., 2004). Figure 7 illustrates a general mechanism of NO pathway in the development of AMS.

Inflammation is a key aspect of our study’s hypotheses. In this study, the neuroinflammation pathway of the individuals who developed AMS was suppressed compared to those that did not develop AMS at SL. This may be a protective mechanism related to acute inflammation stress. In particular, the S100 calcium binding protein (*S100B*) gene exhibited downregulation in AMS + _SL_ compared to AMS─_SL_ individuals prior to their ascent to HA. *S100B* is a marker of blood-brain permeability and is typically increased at HA, especially in those suffering from AMS. Starting with lower levels of *S100B* at SL may protect individuals from AMS when ascending to HA by limiting blood-brain barrier dysfunction at HA. Notably, *S100B* is a recognized biomarker associated with blood-brain barrier dysfunction. Lower levels of *S100B* protein expression in the sublingual region may indicate a heightened susceptibility to blood-brain barrier disruption during hypoxic episodes (Bjursten et al., 2010).

While previous studies have identified *HMGB1*, *TRL4*, *LY96*, *ANGPTL4*, *VEGFA*, and *IL10* as potential inflammation-related markers for AMS, the present study found that only *HMGB1*, *LY96*, and *VEGFA* were detectable, but not significantly differentially expressed. However, this study identified other inflammation markers, such as *S100B, NFκB2*, *STAT2*, and *IL1R2*, as well as a range of neuroinflammation and immune response pathways. The discrepancy in findings could be attributed to several factors, including differences in study design, sample size, participant characteristics, and the specific altitude or ascent profiles used in each study. In Table 4, we summarized transcriptomic responses from on acute mountain sickness (AMS)-susceptible (AMS+) and AMS-resistant (AMS─) individuals to demonstrate the complexity of the landscape.

**TABLE 4:** Transcriptomic responses from 11 research studies on acute mountain sickness (AMS)-susceptible (AMS+) and AMS-resistant (AMS─) individuals ascending from sea level (SL) to high altitude (HA).

Study	Altitude (m)	Subjects (n)	Methods	Study design	AMS + vs. AMS-(DEGs or hub Genes)	AMS pathways regulation	SL vs. HA (DEGs or hub Genes)	Hypoxia Pathways regulation
Chen et al. (2012)	3,250 m, 5,600 m, 4,400 m, 100 m	4 (3 m/1 w)	RNA seq PBMC	25-day expedition to 8,200 m. One blood sample at each altitude. SL blood sample occurred after expedition	NA	NA	*DEGs:* *Up: OSBP2, TMOD1, SIPA1L2, SNCA, HBA1, EPB49, EPB42, DAPK2, SELENBP1, SLC25A3, BLVRB, HBM, ALAS2, ERAF, HBD, HBB, HBG2, HBG1* *Down: LOC, CNN1*	Up: Erythrocyte differentiation, inflammation Down: translation elongation
Goodin et al. (2013)	3,000 m 3,500 m 4,500 m	34	Real-time PCR, Pax Gene whole blood	Acute ascent hypobaric chamber (24 h sample)			*DEGs:* *Up: ADORA2B, ANGPTL4, BTG1, EGR1, F10, F3, HIF1A, HIF1AN, MAP3, NFKB1, PER1, SERPINE1, XNIP, VEGF* *Down: PKM2*	Up: VEGF pathway through ANGPTL4
Liu et al. (2017)	5,300 m, 1 day stop at 3,000 m, 72 h bus ride	10 men	RNA seq PBMC	Staged ascent from 1400 m 72 h blood sample at 5300 m	*DEGs:* *Up: IL13* and *IL17F* *Down: IL2*, *IL4*, *IL6ST*, *IL7*, *IL7R*, *IL10, IL17B, IL32*, and *IL23R* *Hub Genes:* *Up: CCL8, IL17F, DUSP1* *Down: IL10, CCR7, DUSP19*	Up: sprouting angiogenesis, immune responses, inflammatory responses, apoptosis, and erythropoiesis		Up: antigen processing and presentation, cell migration, sprouting angiogenesis, erythropoiesis, and iron homeostasis
Malacrida et al. (2019)	3,830 m	15 (11 m/4 w)	Real-time PCR, 24 h and 72 h samples		NA	NA	*DEGs* *Up: HIF1α, HIF2α, NRF2*	Up: Inflammatory (24 h) and anti-inflammatory pathways (72 h)
Behera et al. (2020)	3,500 m	5 men	Real-time PCR, PBMC	Rapid ascent (2 h sample)	NA	NA	*DEGs:* *Down: Integrin a5, Integrin β1, eNOS*	Down: Cell adhesion
Chang et al. (2020)	3,200 m (Antarctic study)	98 (65 m/33 w)	Microarray, PBMC	Rapid ascent (72 h sample). Previous history AMS, no medication control	All had AMS so no comparisons with non-AMS volunteers	NA	*Hub genes at HA:* *MRPL3, PSMC6, AIMP1, HAT1, DPY30, ATP5L, COX7B, UQCRB, DPM1, and COMMD6*	Up: Protein transport and translation; apoptosis
Gaur et al. (2020)		30 men	RNA seq Pax Gene whole blood	Staged ascent 3 days at 3,100 m prior to ascent to 4111 m (72 h sample)	NA	NA	*Hub genes at HA:* *VIM, CORO1A, CD37, STMN1, RHOC, PDE7B, NELL1, NRP1 and TAGLN*	Up: Antigen processing and presentation, Hematopoietic cell lineage, RAP1 signalling, phagosome 17, viral myocarditis, hypertrophic cardiomyopathy, MAPK signalling Down: Cell adhesion, axon guidance
Pham et al. (2022)	3,800 m	15 (10 m/5 w)	RNA-seq PAX Gene whole blood	Acute ascent (18 h sample)	*10 genes correlated with AMS scores (TNFSF14, FASLG, IL18, CD40LG, PTGER4, MAPKAPK2* *HLADRB1, SMAD7, AGER, MAFK, IRF5)*	Inflammatory biomarker DEGS not associate with AMS severity Dampened immune response in AMS individuals	*DEGs* *Up: BCL2A1, EVI2A, ERGIC2, PPIG, PDCD10, TXNDC9, RGS18, SUB1, TAF7, S100A8, NFXL1, CCDC82, RSL24D1, HMGB1, MAN1A1, NDUFA5, ANKRD12, B2M NORAD, BLOC1S2*	Up: apoptosis signalling, CCKR signalling, ubiquitin proteasome pathway, PDGF signalling, T-cell activation Toll-like receptor (TLR) signalling, RAS and FAS signalling, anti-inflammatory signalling
Li et al. (2023)	3,200 m Meta-analysis (3 data sets)	98 (same data set as Chang et al., 2020 for validation)	Various	Rapid ascent (72 h sample)	NA	NA	*DEGs* *Up:ERH, VBP1, BINP3L, TOMM5, PSMA4, POLR2K* *Down: BINP3L*	Up: HIF-1 signaling pathway, Glycolysis/Gluconeogenesis, amino acid synthesis, carbon metabolism
Kumar et al. (2023)	3,500 m	16 (8 control, 8 Dexamethasone)	72 h blood samples	PBMC, RNAseq	NA	NA	*DEGs* *Up: ID1, CD83, JUN, H2BC18, CXCL8* *Down: CCR2, C-X3-CR1 PBK, CEP55, PSKH2*	Up: Inflammation Down: Cell division, translation or transcription
Yang et al. (2023)	5,260 m (Omics study)	21 (12 m/9 w)	Microarray, PBMC	Acute Ascent (10 h sample)	*10-gene model to predict severe AMS (LL2018 ≥ 6) ACSM1, B4GALT4, DHX58, DIP2B, EPHB3, GALNT3, MARVELD3, OR10G8, OR5B3, RHEBL1*	Up: erythrocyte differentiation, alpha–beta T cell differentiation, and secretion of histamine by mast cells.	*DEGS* *Up: ATP6VOC, CFD, CFP, HMOX1, LGALS1, LILRA2, LILRB2, NEU1, SPI1,P2RX1, VAMP8*	Up Immune function pathways

*DEG, diferentally expressed genes.

Finally, examining the *CREB* pathway in individuals with and without AMS before and after ascent to HA can shed light on its role in the development and progression of AMS. The *CREB* pathway regulates gene expression and is involved in various physiological processes, including learning, stress response, and cell proliferation. The expression of *GPCRs* and growth factor receptor changes between pre- and post-ascent time points, suggests a switch from inhibition to activation of the *CREB* pathway. Hypoxic conditions, are known to activate the *CREB* pathway as cells adapt to low oxygen levels and maintain homeostasis (Nakayama, 2013). Downregulation of *CREB* signaling in susceptible individuals can lead to impaired erythropoiesis and oxygen delivery, making those individuals more vulnerable to AMS; while upregulation of *CREB* after ascent can reflect the body’s response to severe hypoxia stress in AMS individuals.

Recombinant human erythropoietin (rHuEpo) injections mimic the effects of high altitude, particularly in enhancing exercise performance. We compared 45 rHuEpo biomarkers identified by Wang et al. (2017) with the Chamber cohort, which also exercised early during exposure. Of these, 32 markers were detected in our data, with five (*TRIM58, BCL2L1, OSBP2, SNCA, GMPR*) significantly upregulated at high altitude. Notably, *TRIM58* and *OSBP2* were also significantly upregulated at sea level when comparing AMS to non-AMS individuals. *SNCA* plays a critical role in synaptic function and is implicated in neurodegenerative diseases, and *TRIM58* is essential for proper red blood cell development. This serves as an indirect validation of our findings.

Autologous Blood Transfusion (ABT) is another way in sports to elevate hemoglobin, however the transcriptomic study remains limited. In a study by Karanikolou (2023), 49 gene markers were assayed using microarray 10 h after ABT injection. Three DEGs (FLT3, LINC00926, GPR27) were found to overlap with the Chamber cohort, however, the functions of these markers are not very clear.

This study introduces a potential diagnostic biomarker panel and a predictive biomarker for further exploration. Although these biomarkers show promise, their underlying biological mechanisms and associations with AMS require a thorough investigation. Additional data collection efforts at 3,600 m are underway in our laboratory. The validation process will provide valuable information on the diagnostic and predictive capabilities of these biomarkers in the context of AMS, contributing to our understanding of the underlying biological pathways and potentially enhancing our ability to diagnose and predict AMS in the future.

Limitations of our study include the following: 1) the sample size of our cohorts was relatively small, thus requiring replication in the future using larger samples; 2) there may be predisposed genomic or epigenetic factors that could affect gene expression and confound the results of the study 3) AMS occurs when people become hypoxic after ascent to high altitude. Hypoxia alone is known to activate many thousands of genes and proteins. Thus, whether all people experiencing AMS will have a gene expression profile matching those subjects in the present, small study, is unknown and perhaps unlikely. and 4) the Chamber cohort exercised early in the exposure, whereas the other cohort did not which may confound some of the results. 5) This transcriptomic study is based on PBMCs, whose diverse cell types and variable proportions can introduce RNA-seq variability. PBMCs may not capture tissue-specific gene expression changes critical in conditions affecting other organs and are also sensitive to systemic inflammation and stress, which may confound results.

## Conclusion

In conclusion, the identification of a putative transcriptomic signature for AMS is a promising development that could help improve the diagnosis and treatment of this common condition. The gene expression and pathway patterns associated with AMS can help researchers gain a better understanding of the underlying biological mechanisms that contribute to the development of this condition, and potentially develop more targeted and effective therapies.

## Data Availability

The datasets presented in this study can be found in online repositories. The names of the repository/repositories and accession number(s) can be found below: https://www.ncbi.nlm.nih.gov/geo/, GPL11154.
