# Supplementary figures and images for: Transcriptomic signatures of severe acute mountain sickness during rapid ascent to 4,300 m
Source: Front Physiol. 2025 Jan 29;15:1477070. doi: 10.3389/fphys.2024.1477070 (PMC11813865; doi:10.3389/fphys.2024.1477070)

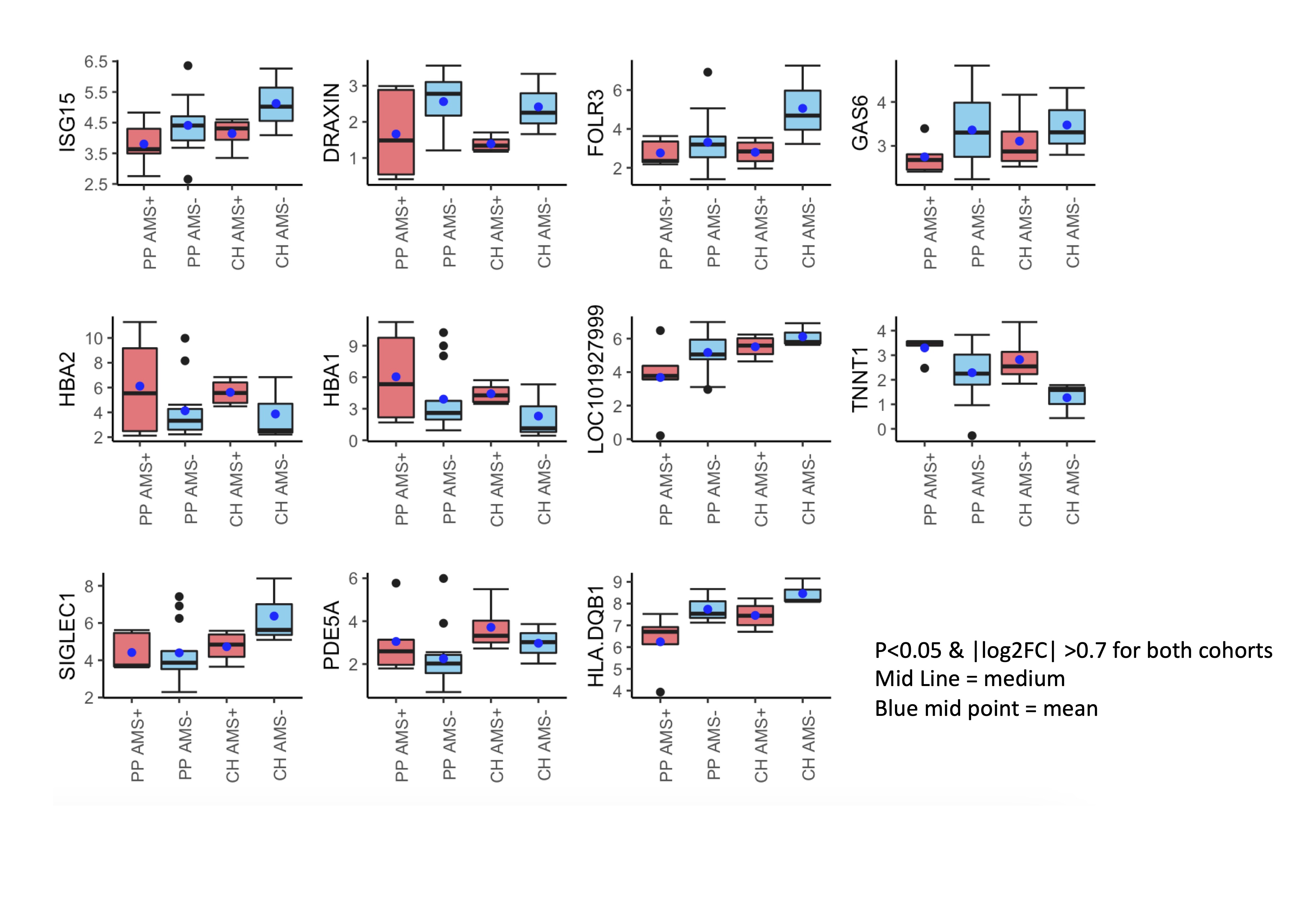

Supplement: Supplementary file 2 [file Image1.jpeg]

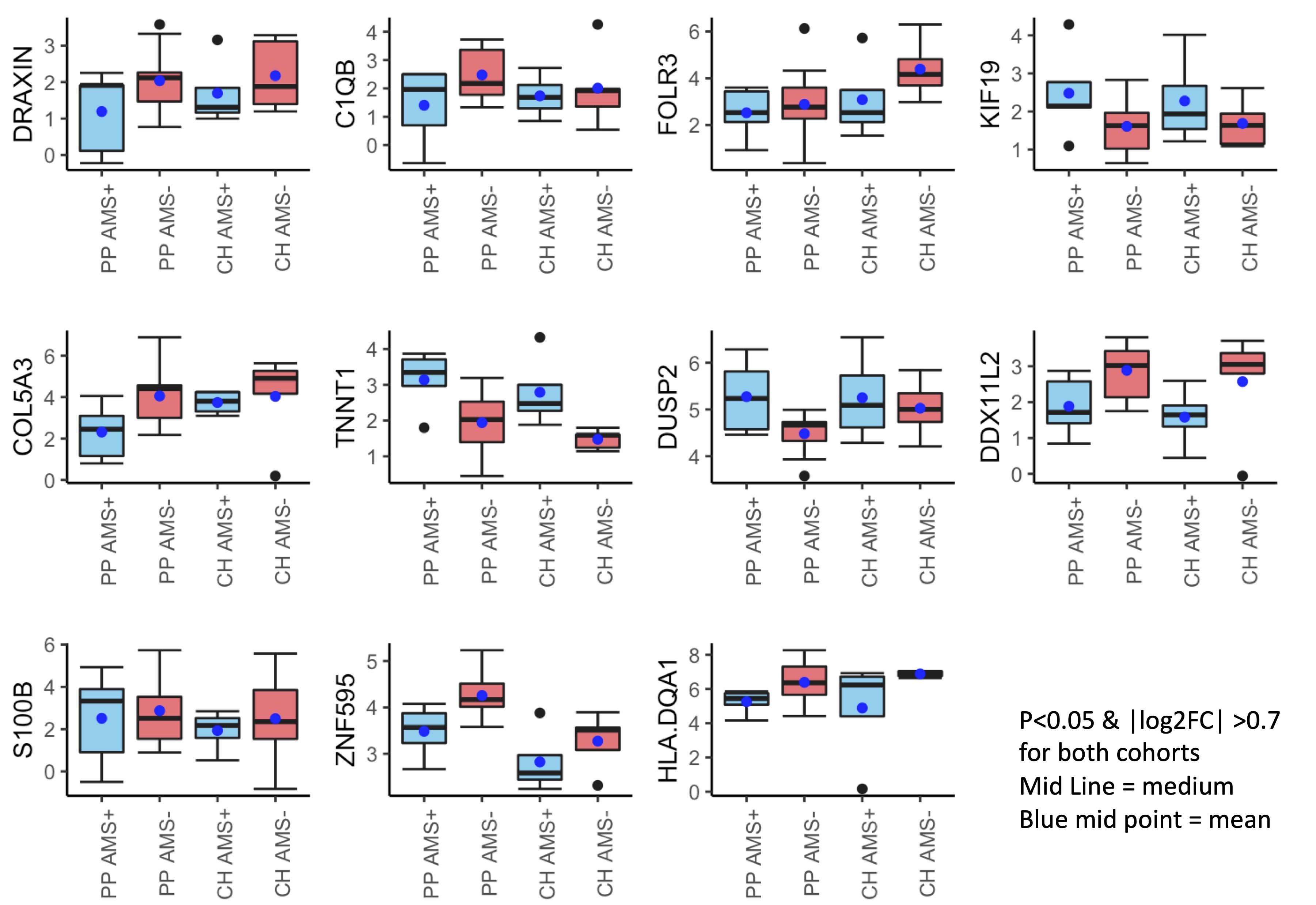

Supplement: Supplementary file 3 [file Image2.jpeg]
