# Supplementary material for: Transcriptomic signatures of severe acute mountain sickness during rapid ascent to 4,300 m
Source: Front Physiol. 2025 Jan 29;15:1477070. doi: 10.3389/fphys.2024.1477070 (PMC11813865; doi:10.3389/fphys.2024.1477070)

**RNA Sequencing Method**

*PBMC separation.* Peripheral blood mononuclear cells (PBMCs) were isolated from 8 mL of venous whole blood samples using Becton Dickinson Cell Preparation Tube (CPT) vacutainers with sodium heparin according to instructions. Vacutainers were inverted 10 times and spun at room temperature (RT) (18 – 25 °C) for 20 min at 1600 RCF in a centrifuge with a horizontal (swing-out) rotor within 1 h of collection. The tubes were spun an additional 5 min, if needed, for adequate cell layer separation. Immediately after centrifugation, about half of the plasma layer was aspirated and discarded without disturbing the ‘foggy’ layer below (containing the PBMCs) using a sterile Pasteur pipette. Without disturbing the separation gel, the cell layer and remaining plasma was then aspirated and transferred to a sterile, RNase free, 15 mL Falcon tube. PBMCs were washed in a 1X Phosphate Buffer Solution (PBS) (made from 9-parts DEPC–treated, nuclease–free, sterile water for RNA work and 1-part molecular grade 10X PBS pH 7.4), by filling the Falcon tube to the 15 mL line and inverting 5 times. The Falcon tube was spun at 300 RCF for 15 min, at RT, in a centrifuge with a horizontal (swing-out) rotor, to insure formation of a pellet. The supernatant was discarded without disturbing the pellet, and the pellet was re-suspended in 12 mL of 1X PBS, using a sterile Pasteur pipette. The Falcon tube was spun at 300 RCF for 10 min, at RT, in a centrifuge with a horizontal (swing-out) rotor (to ensure pellet formation). As much supernatant was aspirated off and discarded without disturbing the pellet. The pellet was re-suspended in 1 mL of pre-aliquoted RNALater solution (Qiagen, Germantown, MD) and placed on ice for 10 min before being frozen and stored in either liquid nitrogen or a -80 degree C freezer.

*RNA-Isolation.* Total RNA was isolated using the PAXgene Blood RNA kit (Qiagen, Valencia, CA) following manufacturer directions. RNA quality and quantity was determined based on Tapestation (Agilent, California) and Nanodrop ND-1000 spectrometer (Thermo Fisher, Wilmington, DE) A260/280 assessments.

*RNA Sequencing.* The total RNA samples were diluted, quantified using the Nanodrop 1000 spectrophotometer (Thermo Scientific, Waltham, MA), and 100ng was used to prepare libraries for sequencing using the TruSeq Stranded mRNA Sample Preparation Kit (Illumina, San Diego, CA) following the manufacturer’s instructions. Briefly, poly-A mRNA molecules wee purified, fragmented, and copied into first strand cDNA. Then the second strand cDNA was synthesized, the 3’ ends were adenylated, single-index adapters were ligated onto the cDNA, and the products were enriched with PCR and purified to create the final cDNA library. The quality of the libraries was determined using the on D1000 ScreenTape on the Agilent TapeStation 2200 (Agilent Technologies, Santa Clara, CA). Libraries were then quantified by qPCR using the KAPA Library Quantification Kit for Illumina Libraries (Kapa Biosystems, Inc., Wilmington, MA) and verified using the dsDNA HS Kit on the Qubit 3.0 Fluorometer (Invitrogen, Carlsbad, CA). Next, the libraries were normalized, pooled, and quantified using the dsDNA HS Kit on the Qubit 3.0 Fluorometer, followed by sequencing 2x150 cycles paired-read on an Illumina HiSeq 4000 platform (Illumina, San Diego, CA) using the Illumina HiSeq 3000/4000 SBS kit following the manufacturer’s instructions. Image analysis and base calling was performed using the Illumina pipeline. Preprocessing of raw base calls, sample de-multiplexing, trimming and filtering was done using the standard procedure of base calling log (bcl) to file sequence output (fastq) conversion.

*Power analysis*

ssizeRNA package was applied with parameters specific to our data for power analysis. The estimated sample size with power 0.8 was 8, which is proximity to our sample size.
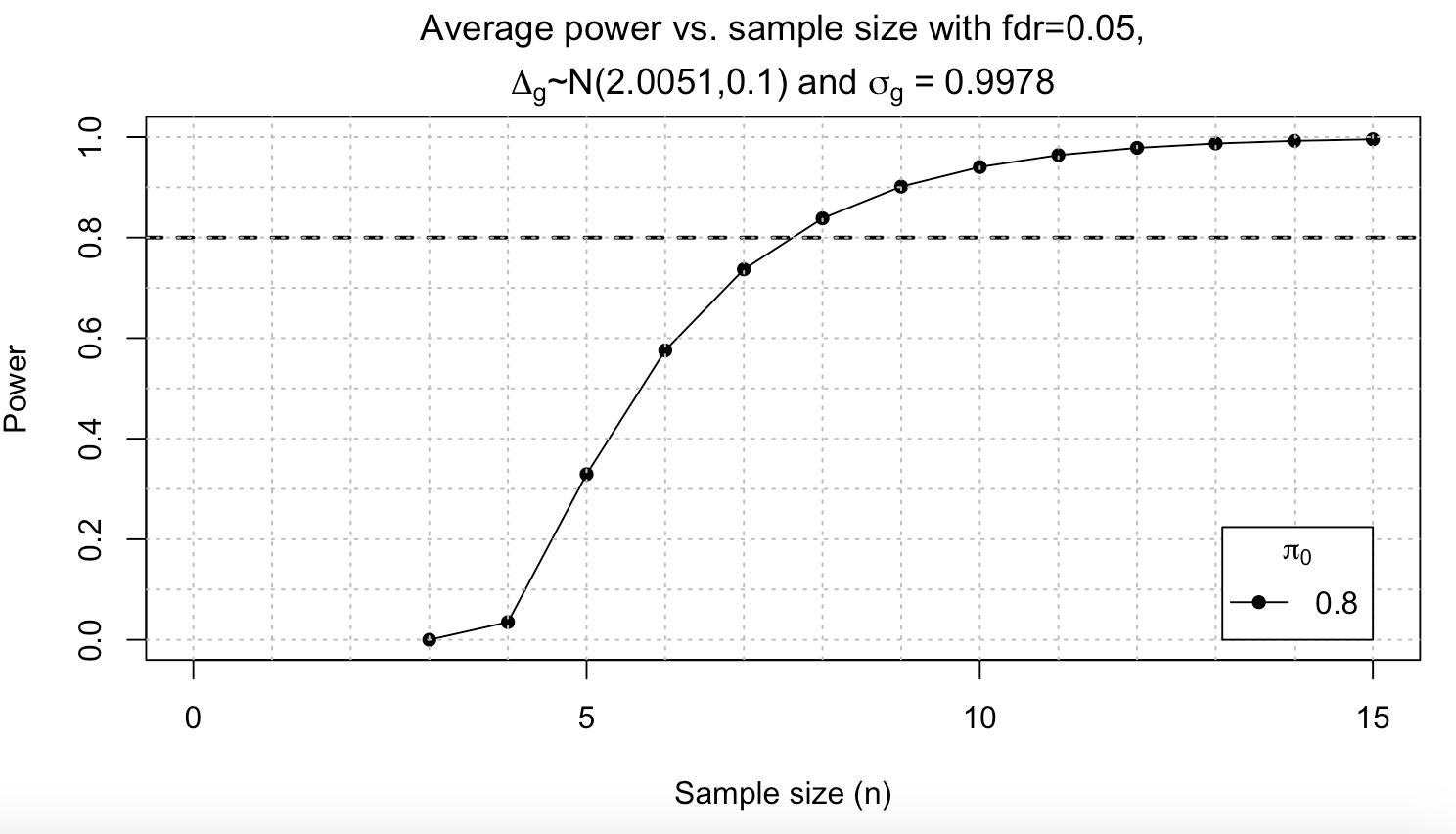

Supplement: Supplementary file 7 [file DataSheet1.docx]
