# Supplementary material for: Transcriptomic signatures of severe acute mountain sickness during rapid ascent to 4,300 m
Source: Front Physiol. 2025 Jan 29;15:1477070. doi: 10.3389/fphys.2024.1477070 (PMC11813865; doi:10.3389/fphys.2024.1477070)
Supplement: Supplementary file 3 [file Supplementaryfile1.docx]

**Table S3.** Common enriched pathways between AMS+$_{HA}$ and AMS+$_{SL}$

| Ingenuity Canonical Pathways | -log(p-value) | z-score | Molecules | -log(p-value) | z-score | Molecules |
|---|---|---|---|---|---|---|
| **cAMP-mediated signaling** | 2.73E+00 | 1.342 | ADCY4,ADRB2,AKAP11,AKAP5,ATF4,CREBBP,CXCR2,DUSP1,FPR2,GDPD3,GNAL,GNAO1,GUCY1A1,HCAR3,LPAR1,OPRL1,P2RY12,PDE1B,PDE5A,PDE7A,PTGDR,PTGIR,SRC,VIPR1 | 2.60E+00 | 0.258 | DUSP1,FPR2,GPER1,GRM2,GUCY1A1,GUCY1B1,P2RY12,P2RY13,P2RY14,PDE5A,PDE9A,PRKAR2B,RGS18,S1PR3,SRC,SSTR3,XCR1 |
| **CREB Signaling in Neurons** | 2.18E+00 | 4.111 | ADCY4,ADGRG1,ADRB2,ATF4,C3AR1,CACNA1A,CACNA2D2,CACNA2D3,CALCRL,CCR2,CMKLR1,CREBBP,CX3CR1,EP300,F2R,FFAR2,FPR2,FZD3,GIPR,GNAL,GNAO1,GNG2,GPR135,GPR162,GPR34,GPR65,GUCY1A1,HCAR3,IGF2R,ITPR1,ITPR2,LPAR1,NMUR1,OPRL1,P2RY1,P2RY12,PDGFRB,PIK3C2A,PLCB1,PLCH2,POLR2H,PTGDR,PTGIR,SOS2,TGFA,TNFRSF11A,VIPR1 | 2.89E+00 | 1.257 | ADGRL1,BMP6,C3AR1,CACNA1H,CACNA2D2,CCR2,CYSLTR1,FPR2,GIPR,GNAZ,GNG11,GPER1,GPR141,GPR25,GPR34,GPR35,GPR65,GRM2,GUCY1A1,GUCY1B1,HGF,P2RY1,P2RY12,P2RY13,P2RY14,P2RY2,P2RY6,PLCD1,PLCG1,PRKAR2B,PRKCZ,RAP1B,S1PR3,SSTR3,XCR1 |
| **G-Protein Coupled Receptor Signaling** | 2.55E+00 | 3.371 | ADCY4,ADGRG1,ADRB2,AMOT,ATF4,C3AR1,CALCRL,CCR2,CMKLR1,CREBBP,CX3CR1,CXCR2,DUSP1,F2R,FFAR2,FOS,FPR2,FZD3,GDPD3,GIPR,GNAL,GNAO1,GNG2,GPR135,GPR162,GPR34,GPR65,GUCY1A1,HCAR3,JUN,KCNN4,LPAR1,MEGF6,MYL9,MYLK,NFKB2,NFKBIA,NMUR1,OPRL1,P2RY1,P2RY12,PDE1B,PDE5A,PDE7A,PIK3C2A,PLCB1,PTGDR,PTGIR,REL,ROCK1,ROCK2,SOS2,SRC,SYNGAP1,VIPR1 | 3.39E+00 | 0.781 | ADGRL1,C3AR1,CCR2,CYSLTR1,DUSP1,FPR2,GIPR,GNAZ,GNG11,GPER1,GPR141,GPR25,GPR34,GPR35,GPR65,GRM2,GUCY1A1,GUCY1B1,HCN2,MEGF6,MYL9,MYLK,NFKB2,P2RY1,P2RY12,P2RY13,P2RY14,P2RY2,P2RY6,PAK4,PDE5A,PDE9A,PLCG1,PRKAR2B,RAP1B,RELB,RGS18,S1PR3,SRC,SSTR3,XCR1 |
| **IL-15 Production** | 3.08E+00 | -0.775 | AATK,EPHA1,EPHA2,EPHB1,EPHB4,FLT3LG,IRF3,JAK3,NFKB2,PDGFRB,PEAK1,REL,SRC,STYK1,TNK1,YES1 | 3.07E+00 | -2.714 | AATK,EPHA1,EPHB4,FLT3LG,KIT,LMTK3,MATK,NFKB2,PRKCZ,RELB,SRC,ZAP70 |
| **Phagosome Formation** | 2.25E+00 | 4.533 | ADGRG1,ADRB2,AP1G2,C3AR1,CALCRL,CCR2,CD209,CMKLR1,CX3CR1,F2R,FCER1A,FCGR1A,FCGR1BP,FFAR2,FPR2,FZD3,GIPR,GPR135,GPR162,GPR34,GPR65,HCAR3,ITGAX,ITGB3,ITPR1,ITPR2,LCAT,LPAR1,MRC1,MSR1,MYL9,MYLK,MYO1A,NMUR1,OPRL1,P2RY1,P2RY12,PIK3C2A,PLA2G4A,PLD4,PTGDR,PTGIR,ROCK1,ROCK2,SOS2,SRC,TLR4,TLR7,TLR8,VIPR1,WASF1,WASL,YES1 | 6.49E+00 | 2.429 | ADGRL1,C3AR1,CCR2,CD36,CLEC4D,CLEC4E,CR2,CYSLTR1,FCGR1B,FCGR2A,FCGR2C,FPR2,GIPR,GPER1,GPR141,GPR25,GPR34,GPR35,GPR65,GRM2,ITGA1,ITGA2B,ITGB3,ITGB5,JCHAIN,LAT,MARCO,MRC2,MSR1,MYL9,MYLK,P2RY1,P2RY12,P2RY13,P2RY14,P2RY2,P2RY6,PAK4,PLB1,PLCG1,PRKCZ,RAP1B,S1PR3,SRC,SSTR3,TLR2,TLR4,TLR5,TLR7,XCR1 |
| **Sperm Motility** | 2.56E+00 | 1.414 | AATK,EPHA1,EPHA2,EPHB1,EPHB4,FLT3LG,GNAL,GNAO1,GNG2,GUCY1A1,ITPR1,ITPR2,JAK3,LCAT,PDE1B,PDGFRB,PEAK1,PLA2G4A,PLCB1,PLCH2,SLC12A2,SRC,STYK1,TNK1,YES1 | 2.97E+00 | 0.378 | AATK,CACNA1H,EPHA1,EPHB4,FLT3LG,GNAZ,GNG11,GUCY1A1,GUCY1B1,KIT,LMTK3,MATK,PLB1,PLCD1,PLCG1,PRKAR2B,PRKCZ,SRC,ZAP70 |
| **Toll-like Receptor Signaling** | 2.09E+00 | 0.447 | FOS,JUN,NFKB2,NFKBIA,PPARA,REL,SIGIRR,TLR4,TLR7,TLR8 | 2.94E+00 | 2.646 | IL18,IRAK3,LY96,NFKB2,RELB,TLR2,TLR4,TLR5,TLR7 |

*Red is upregulated and Green is downregulated
