# Supplementary material for: Transcriptomic signatures of severe acute mountain sickness during rapid ascent to 4,300 m
Source: Front Physiol. 2025 Jan 29;15:1477070. doi: 10.3389/fphys.2024.1477070 (PMC11813865; doi:10.3389/fphys.2024.1477070)
Supplement: Supplementary file 4 [file Supplementaryfile2.docx]

Table S4. Common enriched pathways between AMS–$_{HA}$ and AMS–$_{SL}$

Ingenuity Canonical Pathways	-log(p-value)	z-score	Pikes Peak Molecules	-log(p-value)	z-score	Chamber Molecules
MSP-RON Signaling In Macrophages Pathway	2.28E+00	1.897	CREBBP,HLA-DMB,HLA-DQA1,HLA-DRA,HLA-DRB1,IFNGR2,JUN,PIK3R6,SBNO2,SOCS3	4.49E+00	1.265	CREB5,HLA-DMB,HLA-DOA,HLA-DRB1,HLA-DRB4,IFNGR2,JUN,PTGS2,ST14,TLR4

*Red is upregulated and Green is downregulated
