# Supplementary material for: Transcriptomic signatures of severe acute mountain sickness during rapid ascent to 4,300 m
Source: Front Physiol. 2025 Jan 29;15:1477070. doi: 10.3389/fphys.2024.1477070 (PMC11813865; doi:10.3389/fphys.2024.1477070)
Supplement: Supplementary file 5 [file Supplementaryfile3.docx]

Table S1. Common enriched pathways between AMS+$_{HA}$ and AMS–$_{HA}$

Ingenuity Canonical Pathways	-log(p-value)	z-score	Pikes Peak Molecules	-log(p-value)	z-score	Chamber Molecules
**Activation of IRF by Cytosolic Pattern Recognitior**	2.26E+00	-1.342	CD40,IRF7,ISG15,JUN,NFKB2,REL	2.75E+00	-0.816	DHX58,IFIT2,IRF7,ISG15,STAT1,STAT2
**Crosstalk between Dendritic Cells and Natural Kil**	2.72E+00	-1	CD209,CD40,HLA-A,HLA-C,HLA-F,NFKB2,REL,TLR9	2.03E+00	-1	CD209,CSF2RB,HLA-A,HLA-DRA,HLA-DRB1,HLA-F
**G-Protein Coupled Receptor Signaling**	2.25E+00	1.671	ADGRE3,CALCRL,CNR2,FPR2,FZD3,GNAL,GPR135,GPR171,GPR34,JUN, LPAR1,MEGF6,MYLK,NFAT5,NFKB2,OPRL1,P2RY1,P2RY12,PDE5A,PIK3C2A, PLCB1, PTGDR2,REL,RRAS,S1PR3,S1PR4,SRC,SYNGAP1,XCR1	2.45E+00	0.2	ADGRB1,CREB5,F2R,FICD,FPR2,GNAO1,GNAZ,GNG11,GPER1,GPR15, GPR27,GPR75,GRK5,GUCY1B1,LPAR1,LPAR5,LPAR6,MYL9,MYLK, P2RY2, P2RY6,PDE5A,PRKAR2B,RGS18,TBXA2R
**Macrophage Alternative Activation Signaling Path**	3.77E+00	0.258	ABCA1,CD36,CLEC10A,FCER1A,FCGR2B,HLA-DMA,HLA-DMB,HLA-DPB1, HLA-DQA1,HLA-DQB1,IL1R2,JUN,NFKB2,PIK3C2A,REL	4.28E+00	2.138	CREB5,FCER2,FCGR2B,HLA-DOA,HLA-DQB1,HLA-DRA,HLA-DRB1,IL1B,IL1R2,IRS1,MRC1,PF4,STAT1,THBS1
**Macrophage Classical Activation Signaling Pathw**	2.11E+00	-1.508	BPI,CD40,CXCL10,HIF1A,HLA-DMA,HLA-DMB,HLA-DPB1,HLA-DQA1,HLA-DQB1,NFKB2,TLR9	2.86E+00	-1.508	CXCL10,HLA-DOA,HLA-DQB1,HLA-DRA,HLA-DRB1,IL1B, PARP14,PARP9,STAT1,STAT2,TNFSF13B
**MSP-RON Signaling In Macrophages Pathway**	3.11E+00	1	REL,RRAS,HLA-DMA,HLA-DMB,HLA-DPB1,HLA-DQA1,HLA-DQB1, JUN, NFKB2,PIK3C2A	2.03E+00	0.378	CREB5,HLA-DOA,HLA-DQB1,HLA-DRA,HLA-DRB1,PTGS2,STAT1
**Neuroinflammation Signaling Pathway**	5.30E+00	-2.065	NFAT5,PIK3C2A,TLR8,BIRC6,CD40,CXCL10,HLA-A,HLA-C,HLA-DMA,HLA-DMB,HLA-DPB1,HLA-DQA1,HLA-DQB1,HLA-F,HMOX1,IRF7,JUN, NAIP, NFKB2,PSENEN,REL,S100B,TLR9	2.77E+00	-0.832	BACE2,CREB5,CXCL10,HLA-A,HLA-DOA,HLA-DQB1,HLA-DRA,HLA-DRB1,HLA-F,IL1B,IRF7,PLA2G12A,PTGS2,SNCA,STAT1
**Neutrophil Extracellular Trap Signaling Pathway**	3.94E+00	2.041	PLAAT3,PLCB1,TLR8,BLK,BPI,C1QA,C1QB,CD209,COL18A1,COL6A1,COL6A2, COL9A2,GP1BA,HIF1A,ATP6,CYTB,ND2,ND3,ND4L,NDUFS8,NFKB2,PIK3C2A ,SRC,TOMM7	2.81E+00	0.943	BLK,CD209,COL24A1,COL4A4,COL5A3,COL6A1,FCGR3A,GP1BA,IL1B, LAIR1,ND1,ND6,PF4,PLA2G12A,PLA2G7,SELP,SIGLEC5,SLC25A4
**P2Y Purigenic Receptor Signaling Pathway**	2.22E+00	-0.378	ITGA2B,JUN,NFKB2,P2RY1,P2RY12,PIK3C2A,PLCB1,REL,RRAS	2.31E+00	-0.447	CREB5,GNG11,GUCY1B1,ITGA2B,ITGB3,P2RY2,P2RY6,PRKAR2B
**Phagosome Formation**	4.17E+00	1.521	ADGRE3,CALCRL,CARD9,CD209,CD36,CLEC4D,CNR2,CR1,FCER1A,FCGR2B, FPR2,FZD3,GPR135,GPR171,GPR34,HMOX1,ITGA2B,LPAR1,MYLK,MYO1A, OPRL1,P2RY1,P2RY12,PIK3C2A,PLAAT3,PLD4,PTGDR2,RRAS,S1PR3,S1PR4,SRC,TLR8,TLR9,WASF1,XCR1	2.82E+00	1.569	ADGRB1,CD209,F2R,FCER2,FCGR2B,FCGR3A,FPR2,GPER1,GPR15,GPR27,GPR75,ITGA2B,ITGB3,ITGB5,LPAR1,LPAR5,LPAR6,MARCKS, MRC1, MYL9,MYLK,P2RY2,P2RY6,PLA2G12A,PLA2G7,TBXA2R
**CREB Signaling in Neurons**	1.51E+00	2.558	ADGRE3,CACNA1I,CALCRL,CNR2,FPR2,FZD3,GNAL,GPR135,GPR171,GPR34, HGF,LPAR1,OPRL1,P2RY1,P2RY12,PIK3C2A,PLCB1,PTGDR2,RRAS,S1PR3, S1PR4,TGFA,XCR1	2.63E+00	0.688	ADGRB1,BMP6,CACNA1A,CREB5,F2R,FGFR1,FPR2,GNAO1,GNAZ,GNG11,GPER1,GPR15,GPR27,GPR75,GUCY1B1,LPAR1,LPAR5,LPAR6, P2RY2, P2RY6,PDGFRB,PRKAR2B,TBXA2R

*Red is upregulated and Green is downregulated
