# Supplementary material for: Transcriptomic signatures of severe acute mountain sickness during rapid ascent to 4,300 m
Source: Front Physiol. 2025 Jan 29;15:1477070. doi: 10.3389/fphys.2024.1477070 (PMC11813865; doi:10.3389/fphys.2024.1477070)
Supplement: Supplementary file 6 [file Supplementaryfile4.docx]

Table S2. Common enriched pathways between AMS+$_{SL}$ and AMS–$_{SL}$

Ingenuity Canonical Pathways	-log(p-value)	z-score	Pikes Peak Molecules	-log(p-value)	z-score	Chamber Molecules
CREB Signaling in Neurons	2.19E+00	-3.873	ACKR3,ADGRB2,ADGRG1,BMPR1A,CACNA1I,CACNA2D2,CCR6,CMKLR1,CXCR1,FGFR1,FLT4,GNAL,GPR15,NMUR1,PDGFRB,PTGDR	2.37E+00	-0.408	ADGRD1,BMP6,CACNA1I,CACNA2D3,CALM1,CATSPER1,CYSLTR1,F2R,FFAR2,FGFR1,FLT4,FPR1,FPR2,GNAL,GNAZ,GPR141,GPR68,GRM2,GUCY1B1,LPAR1,LPAR5,LPAR6,PDGFRB,PRKAR2B,S1PR3,TAS1R3,TBXA2R,VEGFA
Crosstalk between Dendritic Cells and Natural Killer Cells	3.72E+00	-1.633	FASLG,HLA-A,HLA-DRA,HLA-DRB1,HLA-DRB4,IL2RB,PRF1	3.77E+00	-2.121	HLA-A,HLA-DRA,HLA-DRB1,HLA-DRB4,HLA-F,IL15,IL2RB,MICA, TLR7,TNFSF10
IL-10 Signaling	6.04E+00	0.577	FCGR2B,FOS,HLA-A,HLA-DQA1,HLA-DQB1,HLA-DRA,HLA-DRB1,HLA-DRB4,IL18RAP,MAF,PRDM1,SOCS3	2.51E+00	0.302	FFAR2,HLA-A,HLA-DMB,HLA-DQA1,HLA-DRA,HLA-DRB1,HLA-DRB4,HLA-F,IFNGR1,IL1RN,STAT1
Macrophage Alternative Activation Signaling Pathway	3.70E+00	0	FASLG,HLA-DQA1,HLA-DQB1,HLA-DRA,HLA-DRB1,HLA-DRB4,MAF,MERTK,SOCS1,SOCS3	5.01E+00	-1.414	CD36,CSF1,FCER1G,FCER2,FPR1,HLA-DMB,HLA-DQA1,HLA-DRA,HLA-DRB1,HLA-DRB4,IL13RA1,IL1RN, JAK2,MARCO, PF4,SOCS1,STAT1,THBS1
MSP-RON Signaling In Macrophages Pathway	5.42E+00	0.632	FOS,HLA-DQA1,HLA-DQB1,HLA-DRA,HLA-DRB1,HLA-DRB4,PTGS2,SOCS1,SOCS3,ST14	2.84E+00	3.162	HLA-DMB,HLA-DQA1,HLA-DRA,HLA-DRB1,HLA-DRB4,IFNGR1,JAK2, PTGS2,SOCS1,STAT1
Natural Killer Cell Signaling	3.54E+00	-1.667	COL18A1,COL5A3,FASLG,FCGR3A,HLA-A, IL12RB2, IL18RAP,IL2RB,KLRB1,LAIR1	3.49E+00	-0.535	COL18A1,COL5A3,FCER1G,FCGR3A,HLA-A,HLA-F,IL12RB2,IL15, IL2RB,JAK2,KLRC1,KLRC3,MICA,NFATC2,TNFSF10
Neuroinflammation Signaling Pathway	2.50E+00	-2.333	FASLG,FOS,HLA-A,HLA-DQA1,HLA-DQB1,HLA-DRA,HLA-DRB1,HLA-DRB4,PTGS2,S100B,SNCA	3.82E+00	-3.153	CASP1,CXCL10,HLA-A,HLA-DMB,HLA-DQA1,HLA-DRA,HLA-DRB1,HLA-DRB4,HLA-F,IFNGR1,JAK2,NAIP,NCF1, NFATC2,PLA2G12A,PTGS2, S100B,STAT1,TLR2,TLR7,TLR8
Pathogen Induced Cytokine Storm Signaling Pathway	9.84E+00	-2.449	CCR6,COL18A1,COL4A3,COL5A3,CXCR1,CXCR2,FASLG,FOS, GZMB,HLA-DQA1,HLA-DQB1,HLA-DRA,HLA-DRB1,HLA-DRB4,IL12RB2,IL18RAP,PF4,PPBP,PRDM1,PRF1,RORC, SOCS3,TBX21,XCL1	9.83E+00	-2.535	AIM2,CASP1,CD163,CLCF1,CLEC7A,COL18A1,COL4A4,COL5A3,COL6A1,CXCL10,CXCL5,CXCR3,DHX58,HLA-DMB,HLA-DQA1,HLA-DRA,HLA-DRB1,HLA-DRB4,IFIH1,IFNGR1,IL12RB2,IL15,IL1RN, JAK2,NLRP6,PF4,PPBP,DDX58,STAT1,TLR2,TLR7,TLR8,TNFSF10,TNFSF13B,VEGFA
Th1 Pathway	6.22E+00	-1.897	HLA-A,HLA-DQA1,HLA-DQB1,HLA-DRA,HLA-DRB1,HLA-DRB4,ICOSLG,IL12RB2,SOCS1,SOCS3,TBX21	7.51E+00	-1.732	CD8A,CXCR3,GATA3,HLA-A,HLA-DMB,HLA-DQA1,HLA-DRA,HLA-DRB1,HLA-DRB4,ICOSLG,IFNGR1,IL12RB2,JAK2, KLRC1,NFATC2, SOCS1, STAT1
Wound Healing Signaling Pathway	2.22E+00	-1	COL18A1,COL4A3,COL5A3,FASLG,FOS,HBEGF,IL18RAP, LAMA2,PF4	2.06E+00	-1.069	CLCF1,COL18A1,COL4A4,COL5A3,COL6A1,IFNGR1,IL15,IL1RN,JAK2, PF4,STAT1,TNFSF10,TNFSF13B,VEGFA

*Red is upregulated and Green is downregulated
